# Global distribution of soil fauna functional groups and their estimated litter consumption across biomes

**DOI:** 10.1038/s41598-022-21563-z

**Published:** 2022-10-17

**Authors:** Petr Heděnec, Juan Jose Jiménez, Jabbar Moradi, Xavier Domene, Davorka Hackenberger, Sebastien Barot, Aline Frossard, Lidia Oktaba, Juliane Filser, Pavel Kindlmann, Jan Frouz

**Affiliations:** 1 Biology Centre ACR, Institute of Soil Biology and Biogeochemistry, Na Sádkách 7, Ceske Budejovice, 37005 Czech Republic; 2 Institute of Tropical Biodiversity and Sustainable Development, University Malaysia Terengganu, 21030 Kuala Nerus, Terengganu Malaysia; 3 Pyrenean Institute of Ecology, IPE-CSIC, Avda, Ntra, Sra, de la Victoria, 16, Jaca, 22700 Huesca, Spain; 4 CREAF- Universitat Autònoma Barcelona, E08193, 08193 Cerdanyola del Vallès, Spain; 5 Department of Biology, Josip Juraj Strossmayer University of Osijek, Cara Hadrijana 8/A, 31000 Osijek, Croatia; 6 IEES-Paris (CNRS, UPMC, IRD, INRA, UPEC), UPMC 4 place Jussieu, 75252 Paris Cedex 05, France; 7 Swiss Federal Research Institute WSL, Zürcherstrasse 111, 8903 Birmensdorf, Switzerland; 8 Soil Science Department, Agriculture Institute, Warsaw, University of Life Sciences-SGGW, Nowoursynowska 166, 02-787 Warsaw, Poland; 9 Department of General and Theoretical Ecology, UFT – Centre for Environmental Research and Sustainable Technology, University of Bremen, FB 02, Leobener Straße 6, 28359 Bremen, Germany; 10 Faculty of Science, Institute for Environmental Studies, Charles University, Benátská 2, Praha, 12800 Czech Republic

**Keywords:** Ecology, Ecology

## Abstract

Soil invertebrates (i.e., soil fauna) are important drivers of many key processes in soils including soil aggregate formation, water retention, and soil organic matter transformation. Many soil fauna groups directly or indirectly participate in litter consumption. However, the quantity of litter consumed by major faunal groups across biomes remains unknown. To estimate this quantity, we reviewed > 1000 observations from 70 studies that determined the biomass of soil fauna across various biomes and 200 observations from 44 studies on litter consumption by soil fauna. To compare litter consumption with annual litterfall, we analyzed 692 observations from 24 litterfall studies and 183 observations from 28 litter stock studies. The biomass of faunal groups was highest in temperate grasslands and then decreased in the following order: boreal forest > temperate forest > tropical grassland > tundra > tropical forest > Mediterranean ecosystems > desert and semidesert. Tropical grasslands, desert biomes, and Mediterranean ecosystems were dominated by termites. Temperate grasslands were dominated by omnivores, while temperate forests were dominated by earthworms. On average, estimated litter consumption (relative to total litter input) ranged from a low of 14.9% in deserts to a high of 100.4% in temperate grassland. Litter consumption by soil fauna was greater in grasslands than in forests. This is the first study to estimate the effect of different soil fauna groups on litter consumption and related processes at global scale.

## Introduction

Soils host diverse organisms including invertebrate fauna that greatly increase the global turnover of dead organic matter^1–4^. Despite their small size, soil animals provide key ecosystem processes such as the decomposition of organic matter and the recycling of nutrients^3,5–7^. Soil animals are classified according to their body size into microfauna (< 0.2 mm), mesofauna (> 0.2 mm), and macrofauna (> 2 mm)^5,8–10^. Microfauna are mostly predators of soil bacteria and fungi (e.g. protists and some nematodes), but some groups of soil microfauna are saprophagous and contribute to litter decomposition (e.g., litter-feeding nematodes)^11–13^. Macro- and mesofauna are mostly saprophagous whereas some mesofauna such as collembolans or macrofauna such as dipteran larvae function distinctly as fungal and bacterial feeders, respectively^6–8^. In addition, feeding activity of soil macro- and meso-fauna alter environmental conditions in topsoil and thus shape composition and diversity of soil microorganisms^5,14–22^. Soil macrofauna such as earthworms particularly contribute to bioturbation and formation of soil aggregates^3,5^. Predacious arthropods such as centipedes, spiders, and predacious ants indirectly enhance litter decomposition by altering patterns of cascading effects on lower trophic levels via reducing competition and resource overexploitation^23^. Finally, subsurface herbivores such as herbivorous nematodes or insect larvae directly shape communities of aboveground vegetation which in turn structure subsurface communities via litter input^1,3,5^. Table 1 gives a detailed list of soil fauna functional groups considered in this study. Despite significant progress in research focused on soil fauna, the interrelated functional roles of various groups of soil fauna remain poorly understood^24^.

**Table 1:** Main classification of soil fauna functional groups with bold terms used in text.

Trophic level	Functional level	Taxonomic level
**Herbivores**		
**Omnivores**	Other	
Omnivores		**Ants**
**Predators**		
**Saprotrophs**	Other	
Saprotrophs	**Bacterial feeders**	
Saprotrophs	**Fungal feeders**	
Saprotrophs	**Litter feeders mesofauna**	
Saprotrophs	**Litter feeders macrofauna**	Other
Saprotrophs	Litter feeders macrofauna	**Earthworms**
Saprotrophs	Litter feeders macrofauna	**Termites**

Soil fauna functional groups: *Bacterial feeders* Protists, bacterivorous nematodes, dipteran larvae, rotifers, tardigrades. *Fungal feeders* Oribatid mites, collembolans, pauropods, proturans, fungivorous nematodes. *Herbivores* Gastropods, hemipterans, homopterans, herbivorous nematodes. *Litter feeding macrofauna* Unidentified soil fauna larger than 2 mm. *Litter feeding macrofauna* Unidentified soil fauna smaller than 2 mm. *Omnivores* Blattodea, Dermaptera, myriapods sensu lato*,* omnivorous nematodes, insects sensu lato*. Predators* Spiders, centipedes, Mesostigmats, Diplura. *Saprotrophs* Saprotrophic nematodes, isopods, milipedes, enchytreids.

Soil fauna assimilate only part of consumed litter and return undigested litter fragments to the soil in the form of faeces^25^. The defecation and incorporation of faeces in the soil by faunal activity (for example bioturbation) change the physical–chemical properties of organic matter^7,26,27^. These changes contribute to the stabilization of soil organic matter and the formation of soil aggregates, which indirectly affect soil water content, nutrient storage, and ion exchange capacity^3,17^. The global estimation of litter amount which is being processed by soil fauna is unknown, but local studies indicate that more than 50% of net primary production (NPP) is returned to the soil via litter consumption by various groups of soil fauna^3,28,29^. The quantity of litter consumed, however, is likely to vary across biomes. Processing of litter by soil fauna significantly affects its physical–chemical properties^17,30–34^. Many studies^3,5,17,25,29^ have also reported that soil fauna promote the decomposition of leaf litter via their direct effect on litter fragmentation and comminution, which in turn facilitate the colonization of litter by soil microorganisms. Despite the substantial evidence that soil fauna greatly affected litter transformation, the quantitative estimates of faunal biomass as well as of litter consumption by different faunal groups remains poorly understood.

It is important to distinguish between the effect of fauna on decomposition *vs.* the quantity of litter consumed by fauna^35^. Litter consumption by soil fauna and transformation of litter into faeces affect physical and chemical soil properties such as water holding capacity and soil pH^21,36^. Accumulation of faunal faeces in soil horizons thus feeds back into formation of soil aggregates, alteration of plant communities, mycorrhiza association, and/or decomposer food web composition^21,36,37^. However, empirical constraints on the amount of litter consumed by soil fauna are needed to improve understanding of the effects of soil fauna not only on litter decomposition but also on many other key ecological processes such as nutrient cycling and organic matter transformation^3,38,39^. Many researchers^16,30,40,41^ have measured the effect of soil fauna on decomposition by employing an “input *vs* output” approach using multifactorial treatments with and without fauna, specifically use litterbags to exclude or include soil fauna of a particular size. In such experiments, decomposition is usually defined as mass loss from the litterbags. However, mechanisms controlling mass loss from litterbags remain uncertain. Litter mass loss can occur due to increased microbial mineralization or by leaching and washing out of small organic matter fragments. Litter consumption by soil fauna may either accelerate or slow-down microbial mineralization of fauna-processed litter^3^. However, as noted above, the litter consumption and litter transformation of litter into faeces affects not only litter mineralization but also habitats for soil microbiota and plant roots. This research thus addresses the overall amount of litter which is consumed by fauna rather than differences between litter decomposition with and without fauna. We anticipated the quantity of litter consumed by fauna to exceed the final net effect of fauna on decomposition, which is often calculated as the difference in litter mass loss between fauna-accessible and non-accessible litterbags^40^ (see Supplementary Method S1). This research demonstrates that integrated data on feeding activity and biomass of different functional groups of soil fauna across various biomes can effectively quantify the amount of fauna-consumed litter and thus estimate faunal effects on various soil processes at larger scales.

The global distribution of soil fauna and their consumption of litter is interpreted to depend on a set of hierarchical factors like climate, soil properties, and vegetation, which are often themselves interrelated^19,24,28,42^. Climate modifies litter decomposition indirectly via its effects on vegetation, soil type, and soil organismic communities^19,28,43,44^. For example, a global study by Wall et al.^19^ reported that soil animals increased decomposition rates in temperate and wet tropical climates but exerted neutral effects where temperature or moisture constrain biological activity. Soil physico-chemical properties such as moisture and pH influence biomass of soil bacteria and fungi. These in turn provide food resources for microbial and detritovore communities^45^. The chemical composition of leaf litter and especially its C:N ratio and lignin content significantly influence soil fauna biomass and community structure^46–48^. For example, decomposition rates decrease with increased C:N ratio in litter but increase with mean annual temperature and precipitation^22^. This is reflected also by decomposability of litter, where grass litter decomposes more easily than broadleaf litter which decomposes more easily than coniferous litter^49,50^. In recent decades, extensive research efforts have sought to estimate the global diversity of soil organisms^12,51,52^, and published data now provide quantitative data on the distribution of biomass of major faunal groups. Combining this data with data on the rate at which different soil fauna consume litter allows for estimation of litter consumption rates among different biomes and even on global scales. We can also now estimate which factors associate with higher and lower rates of litter consumption by soil fauna.

The present global-scale study had three main objectives: (1) to estimate soil fauna functional group biomass among biomes, (2) to estimate litter consumption by saprotrophs and belowground herbivores among biomes, and (3) to identify major factors related to differences in faunal biomass and litter consumption among biomes. Some groups of soil fauna may also feed on living plant matter, so we also estimated herbivory of belowground plant tissue by soil fauna, as this might impact the overall pattern^53^. In addition, we assessed the potential effects of predators that exert top-down control on detritivores and that thereby affect litter comminution and organic matter decomposition rates^23^. We hypothesized that the biomass of soil fauna functional groups and litter consumption depends on climatic factors and will be higher in warmer and wetter biomes relative to drier and colder biomes. The proportion of annual litterfall consumed by soil fauna likely depends on litter quality. We therefore hypothesized that the proportion of annual litter fall consumed by fauna would follow patterns of litter decomposability with the highest proportions in grass-dominated biomes such as temperate and tropical grasslands followed by biomes dominated by broadleaf and then coniferous flora.

## Results

### Distribution and biomass of functional groups across biomes

**Figure 1:**
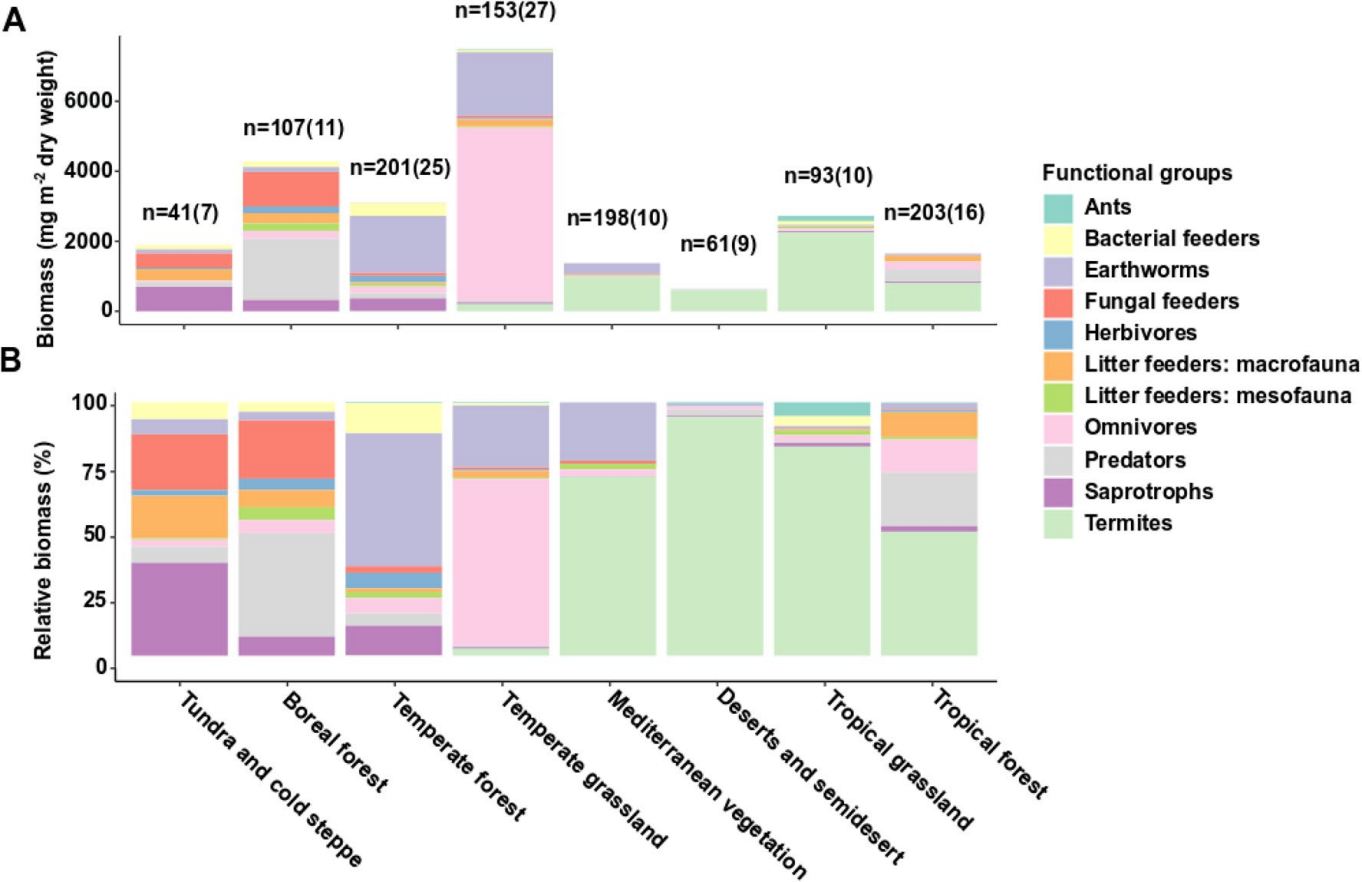
Global distribution among biomes of the biomass (**A**) and the relative biomass (**B**) of soil fauna functional groups. The n refers to number of observations based on individual habitat studies. Number in parentheses refers to number of published studies.

A quantitative review of the literature showed that total soil fauna biomass and its distribution among functional groups varies among biomes (Fig. 1A, Table S1). The biomass was highest in temperate grasslands and then decreased in the following order: boreal forest > temperate forest > tropical grassland > tundra > tropical forest > Mediterranean ecosystems > desert and semidesert (Fig. 1A). Although omnivores, termites, earthworms, litter-feeding fauna, and/or predators generally represented the dominant groups in terms of biomass, this was strongly biome-dependent (Supplementary Table S1). Tropical grasslands, desert biomes, and Mediterranean ecosystems were dominated by termites (Fig. 1B). In contrast, temperate grasslands were dominated by omnivores, while temperate forests were dominated by earthworms. Biomass of predators was highest in taiga forests, and saprotrophs were the dominant group in tundra and cold steppes (Supplementary Table S1).

### Climate and litter quality shape biomass and distribution of soil fauna functional groups

**Figure 2:**
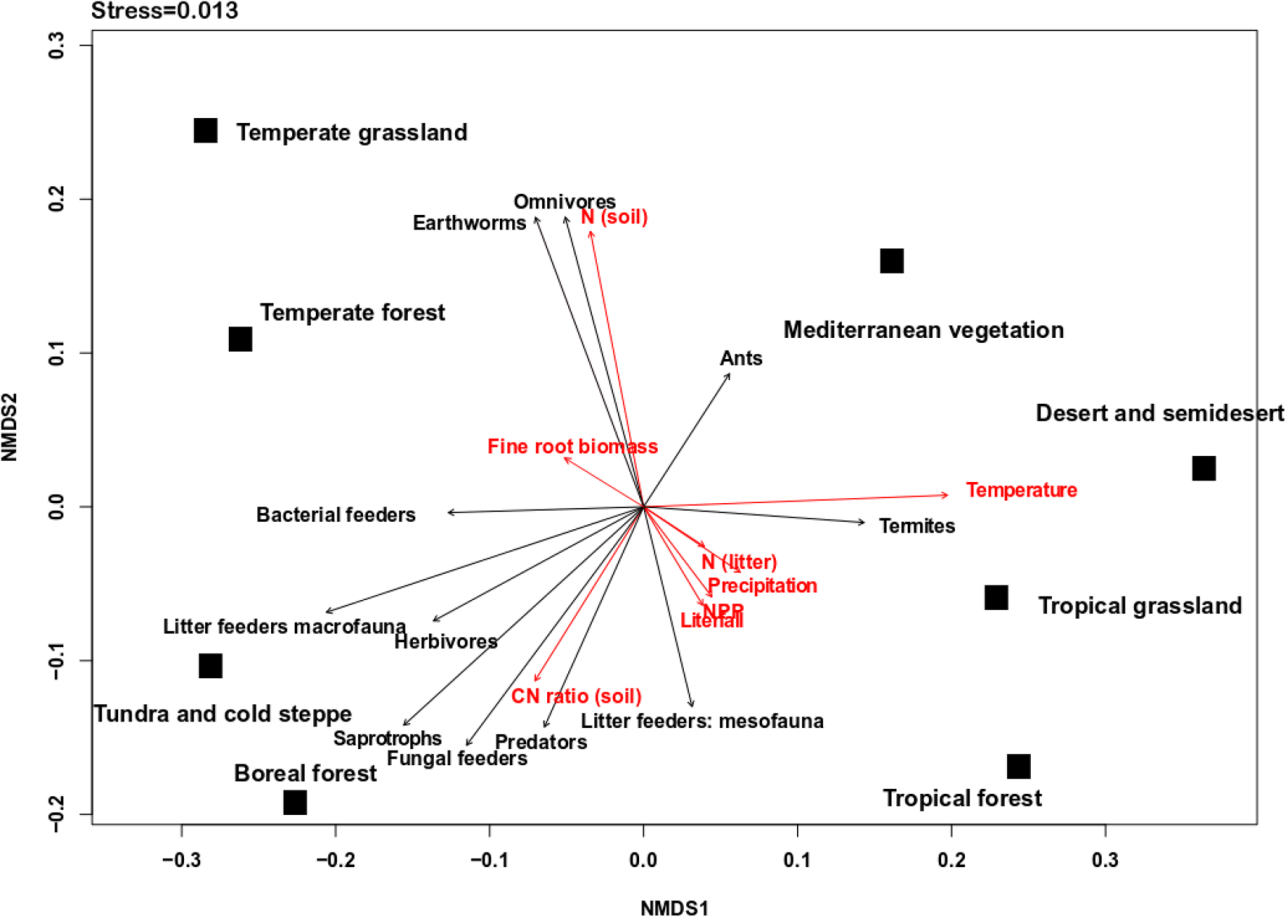
Non-metric multidimensional scaling (NMDS) of climatic factors, litter quality, and biomass of soil fauna functional groups. Red arrows indicate climatic factors and litter quality. Black arrows indicate biomass of soil fauna functional groups. NMDS procedure computed configuration in 999 iterations with a stress value of 0.013.

**Figure 3:**
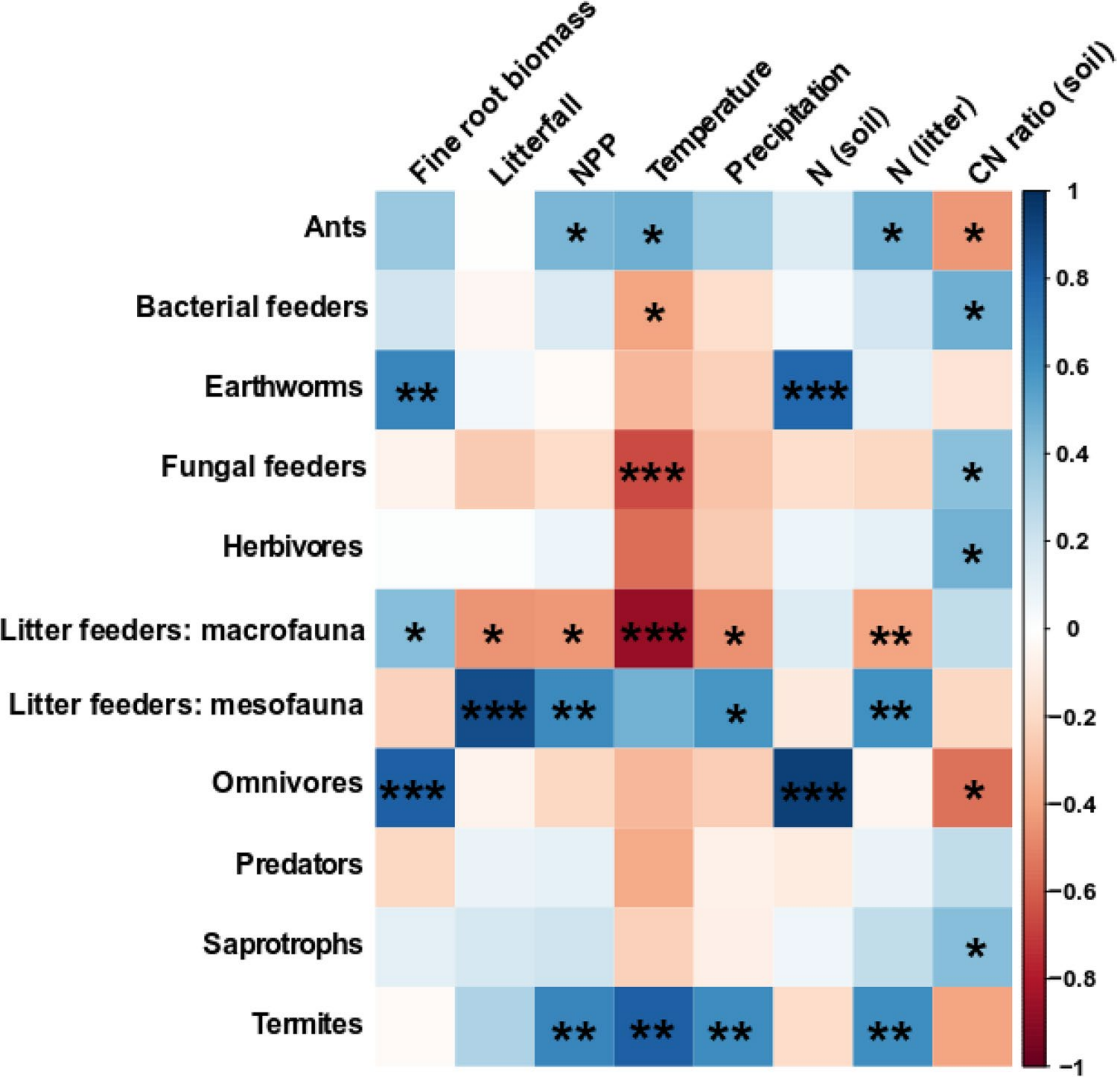
Heatmap of Pearson’s correlation coefficients for the relationships between the biomass of soil fauna by functional group and the following variables: fine root biomass, litterfall, net primary production (NPP), mean annual temperature (MAT), mean annual precipitation (MAP), N content of soil, N content of litter, and the litter C:N ratio. Asterisks indicate significant correlations: *, **, and *** indicate p < 0.05, < 0.01, and < 0.001, respectively. Bonferoni corrections were used to adjust p value of multiple correlations. Blue color indicates positive correlation. Red color indicates negative correlation.

**Figure 4:**
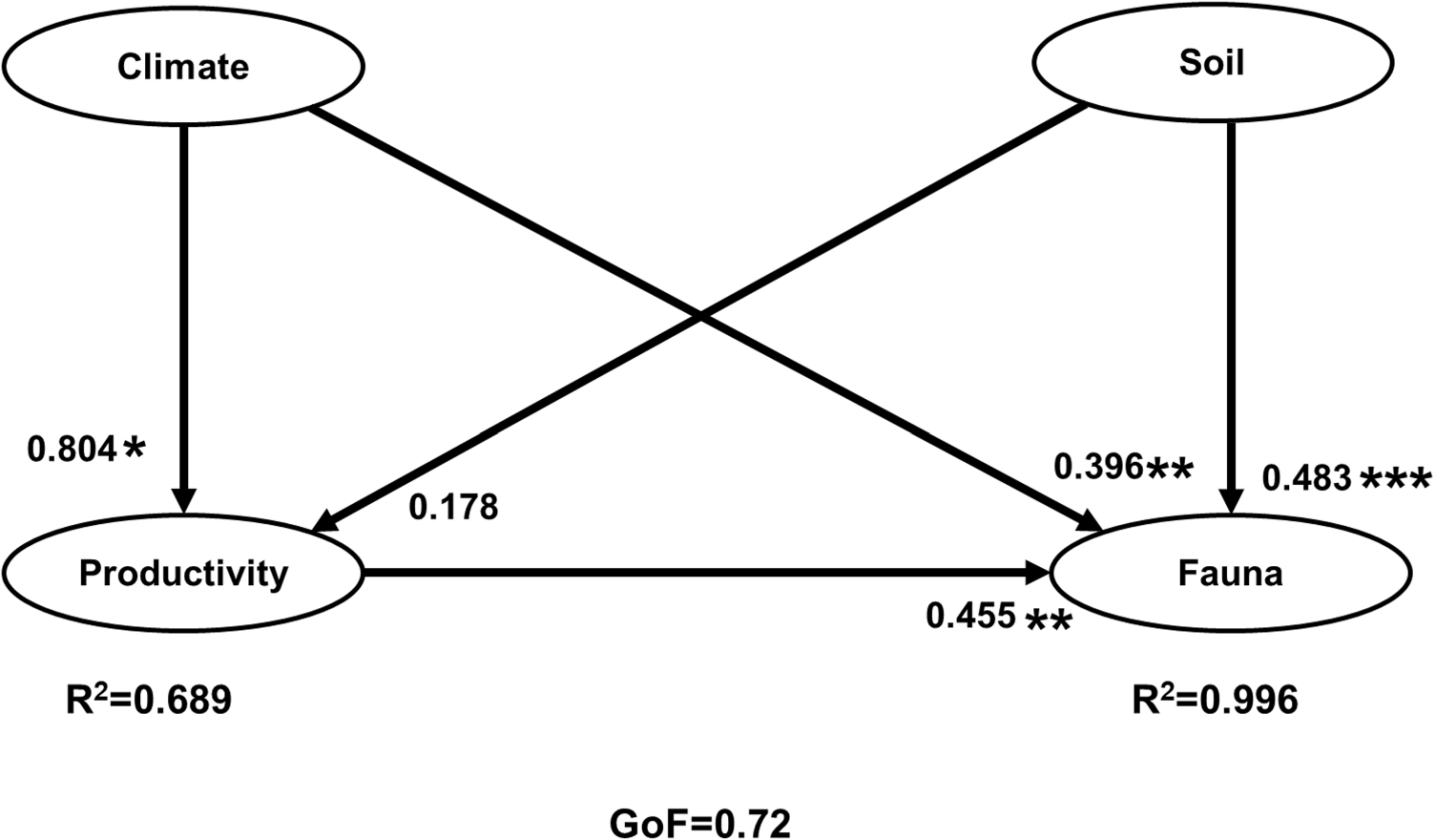
Path model based on the direct effects of climate (MAT, MAP), soil (soil N and C:N ratio), and productivity (NPP, N (litter) and mean annual litterfall) on biomass of soil fauna functional groups associated with litter transformation (earthworms, termites, saprotrophs, herbivores, litter feeding mesofauna and macrofauna). Solid lines indicate direct effects. Goodness of Fit (GoF) = 0.72. Asterisks indicate significant effect: *, **, and *** indicate p < 0.05, < 0.01, and < 0.001, respectively.

The NMDS analyses indicated that environmental factors closely associated with soil fauna biomass differed among biomes. Soil faunal biomass was most closely associated with the litter C:N ratio in tundra and boreal forests. Tropical biome data showed close association between soil fauna biomass, temperature, precipitation, and NPP (Fig. 2). In temperate biomes, faunal biomass was most closely associated with soil N availability (Fig. 2). Mean annual temperature, precipitation, mean annual litterfall, fine root biomass, and litter chemistry (N content in litter) significantly affected the biomass of soil fauna functional groups (Fig. 3). For example, biomass of herbivores (soil fauna that consume roots and other living, belowground plant tissues), fungal feeders, bacterial feeders, and litter-feeding macrofauna decreased with mean annual temperature while biomass of termites and ants increased with mean annual temperature (Fig. 3). Functional groups associated with litter chemistry (N content in litter) were ants, termites, herbivores, and saprotrophs. In summary, statistical analyses (PLS-PM) indicated positive effects of climate, soil chemistry, and plant productivity on soil fauna functional group biomass (Fig. 4).

### Litterfall and litter consumption across biomes

**Figure 5:**
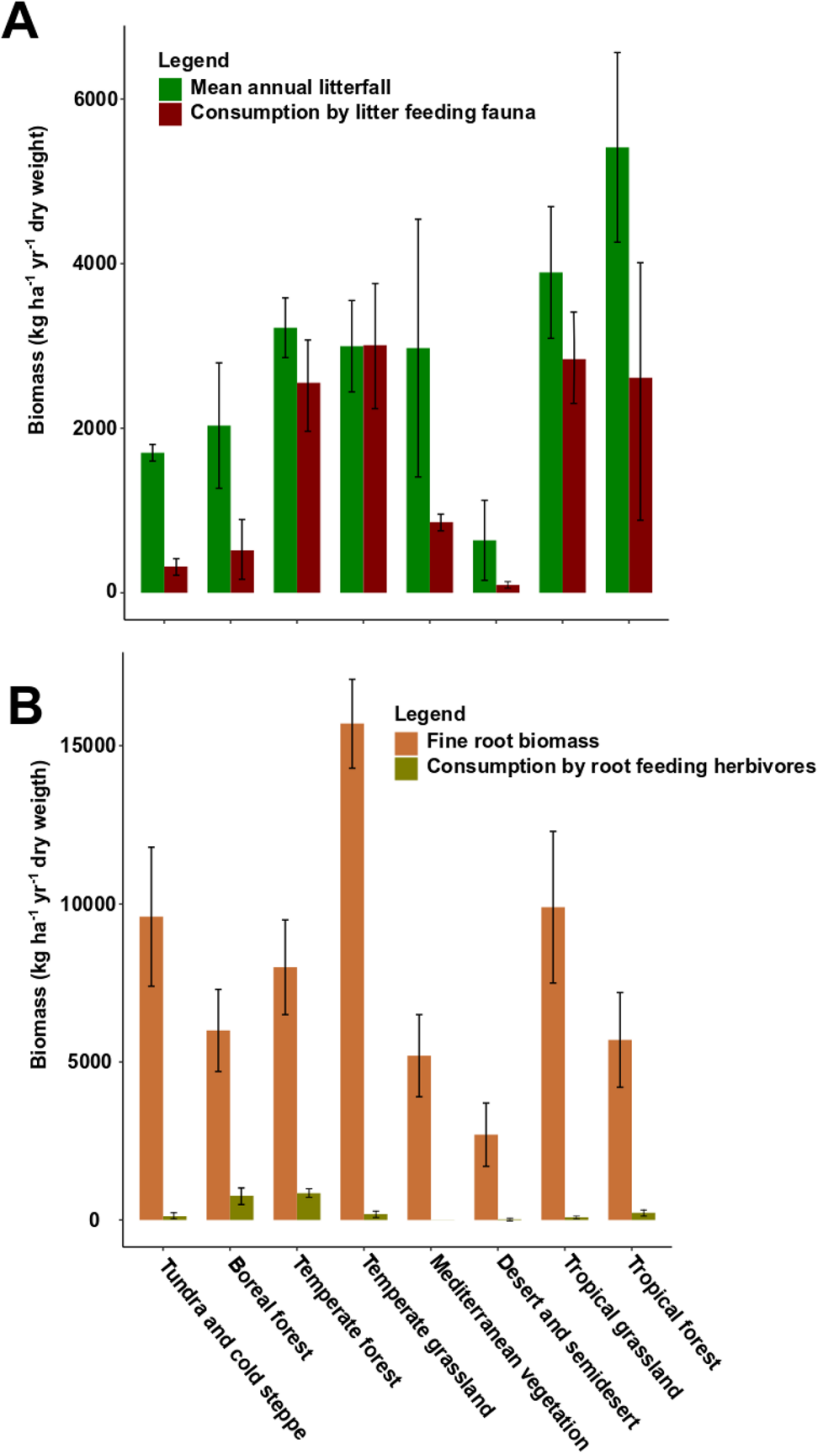
Mean annual litterfall and estimated consumption of litterfall by soil fauna across biomes (**A**) and estimated quantity of belowground living fine roots consumed by belowground herbivores across biomes (**B**). Values for annual litterfall in (**A**) and fine root biomass (**B**) are means ± SE.

The mean annual litterfall varied across biomes (Fig. 5A,B, Supplementary Table S2). Annual litterfall was higher in forests than in grasslands or Mediterranean plant communities and was lowest in tundra and deserts. Mean litter stock shows similar patterns with higher litter stock in forests and lower litter stock in grasslands and deserts. The fine root biomass was high in temperate grassland, tropical grassland, and tundra but low in the temperate forests, tropical forest, boreal forest, and Mediterranean biomes. Similar to annual litterfall, deserts and semideserts showed the lowest fine root biomass.

The biomass data shown in Fig. 1A,B and data on food consumption rates of major faunal groups (Table 2) were combined to estimate the potential amount of litter consumed by soil fauna (Fig. 5A,B, Table 2). The estimated quantity of litter consumed by soil fauna was highest in temperate biomes, followed by tropical biomes and taiga, and lowest in Mediterranean biomes, tundra, and deserts. Belowground herbivores consumed significantly lower biomass of fine roots than soil fauna consumed litter. Boreal and tropical forests exhibited the highest consumption of fine roots by belowground herbivores, while Mediterranean biomes and deserts showed the lowest consumption of fine root biomass by belowground herbivores.

**Table 2:** Mean annual litterfall, percentage of litter consumed by soil fauna, biomass of fine roots, percentage of fine roots consumed by belowground herbivores, turnover rate and turnover time as affected by biome.

	Mean annual litterfall (kg ha^−1^ year^−1^)	Annual litter stock (kg ha^−1^)	Percentage of litter consumed by litter feeders (kg ha^−1^ year^−1^)	Fine root biomass (kg ha^−1^)	Percentage of fine roots consumed by belowground herbovores	Turnover rate
Tundra and cold steppe	1702 ± 706	5210 ± 2946	18.8	9600 ± 2200	1.3	0.327 ± 0.24
Boreal forest	2032 ± 1094	8925 ± 4758	25.4	6000 ± 1300	12.8	0.228 ± 0.23
Temperate forest	3221 ± 1394	11,112 ± 6345	79.2	8000 ± 1500	1.6	0.29 ± 0.22
Temperate grassland	2997 ± 1053	2500 ± 707	100.4	15,700 ± 1400	1.2	1.199 ± 1.489
Mediterranean vegetation	2974 ± 1480	5318 ± 3090	28.9	5200 ± 1300	0.1	0.559 ± 0.479
Desert	638 ± 448	918 ± 698	14.9	2700 ± 1000	0.8	0.695 ± 0.642
Tropical grassland	3893 ± 1894	3378 ± 1990	72.9	9900 ± 2400	0.9	1.152 ± 0.952
Tropical forest	5413 ± 1394	6300 ± 3366	48.3	5700 ± 1500	4	0.859 ± 0.414
Average			48.6		3.9	

Values refer to mean ± SE.

Mean annual litterfall refers to leaf litter biomass produced during 1 year.

Percentage of leaf litter consumed by litter feeders refers to litter consumed by litter feeders during 1 year.

Fine root biomass refers to biomass of fine roots (≤ 2 mm in diameter) over 0–30 cm depth.

Percentage of fine roots consumed by belowground herbivores over 1 year.

Turnover rate refers the ratio of mean annual litterfall and mean litter stock.

References for this table are included in Table S2.

On average across all biomes, soil fauna consumed about 50% of annual litterfall, but this percentage ranged from 14.9% in deserts and semideserts to 100.4% in temperate grasslands. By contrast, belowground herbivores consumed only 4% of fine root biomass (Table 2). The percentage of litterfall consumed was highest in temperate and tropical grasslands followed by temperate forest and tropical forest. Mediterranean biomes, boreal forest, tundra, and deserts exhibited lower litter consumption. Similarly, the highest turnover rates (ratio of mean annual litterfall to litter stock) appeared in grassland biomes and tropical forests, while boreal forests and tundra showed the lowest turnover rates. The highest turnover time (ratio of litter stock to mean annual litterfall) appeared in boreal forest, temperate forest, and tundra (Supplementary Table S2). Grassland biomes showed the lowest turnover time (Supplementary Table S2).

## Discussion

### Potential soil fauna litter consumption across biomes

On average across all biomes, fauna consumed 48.6% of the annual litterfall, an estimate that agrees with litter decomposition reported by García-Palacios^22^ and Wardle^28^. Large differences appeared among biomes however with higher consumption in tropical and temperate biomes relative to that in colder or arid biomes. These observations agree with a meta-analysis of litterbag studies, which detected the largest faunal effects on litter decomposition in a continental temperate climate^30^. Our results are also consistent with a global assessment of faunal decomposition activity by Wall et al.^19^, who found that decomposition rates varied across climatic zones and biomes.

In temperate regions as well as in the tropics, our review of available literature found that litter consumption appeared higher in grasslands than in forests. Overall, fauna appear to consume more litter in biomes dominated by herbs and grasses, which produce litter with lower C:N ratios and lower lignin content. Generally lower consumption rates appear in biomes dominated by trees, which produce litter with higher C:N ratios and higher lignin content^54,55^. In addition, global meta-analyses by Pietsch et al.^55^ demonstrated that the gymnosperms (conifers) exhibited significantly lower decomposition rates than angiosperms. Our study found that among the colder biomes, faunal litter consumption was higher in conifer-dominated boreal forests than in herbal vegetation-dominated tundra. Elevated levels of faunal consumption of annual litter in tundra and boreal forests contrasts that reported in Wall et al.^19^, a study which found no faunal effects in these biomes. Our study also considered litter stock, which can indicate decomposition activity across biomes. Grassland biomes showed lower litter stock than forest biomes but relatively high mean annual litterfall. This indicates that grassland biomes with high quality leaf litter experience higher decomposition rates than forest biomes^19,56^. We also calculated turnover rates per each biome (mean annual litterfall divided by litter stock) and turnover time as ratio of litter stock and mean annual litterfall. Both indices showed higher turnover rate and faster turnover time for grassland biomes than forest biomes. In addition, cold biomes showed lower turnover rates and longer turnover time than warmer biomes.

The finding that litter quality is a major factor determining litter consumption corresponds well with the expected effects of the associated faunal groups on the formation of humus and its distribution in major world ecosystems^3,57^. In colder biomes dominated by vegetation that produce litter with high C:N ratios, the litter consumption is dominated by macro- and mesofauna, which fragment litter and convert it into the faeces that accumulate on soil surface to form a mor-type humus with plant residual in varying states of decomposition^28,56,57^. By contrast, temperate biomes exhibit a higher proportion of earthworms and greater bioturbation. These traits typify modern humus which contains partially decomposed residues of broad-leaf deciduous trees or mull humus having deeper A horizons with well-decomposed organic matter mixed deeply into the mineral soil^57–59^. We therefore suggest that the effects of fauna on humus properties depend on the litter C:N ratio. Earthworm contributions and bioturbation increase as the litter C:N ratio decreases. In tropical biomes, termites play a dominant role in litter processing. Their contribution seems to increase with habitat aridity. Termites are known as ecosystem engineers^60^ and may also contribute to soil mixing and bioturbation^57,60,61^.

As expected, the present study found that fauna consume more litter than indicated by studies using litterbags and similar methods^30^. The method used in the present study determined consumption by multiplying biomass by consumption rate while the litterbag method attempts to measure different quantities. The former method estimates the proportion of annual litterfall consumed by soil fauna while litterbags seek to determine how the presence or absence of fauna influence microbial decomposition of litter. If fauna consume 100% of the annual litterfall and decompose it by 50%, the soil fauna contribution to litter decomposition would be the difference between decomposition in fauna-accessible and non-accessible litterbags, which would be 50% if no litter decompose in non-accessible litterbags, but likely less as some litter usually decompose there. The higher will be decomposition in fauna non-accessible litterbags the lover will appear fauna effect (see Supplementary Method S1). These scenarios may explain why an extensive meta-analysis by Frouz et al.^30^ found no significant effect of fauna on decomposition of ‘high quality’ litter (i.e., with C:N ratios ≤ 20). While field studies^18,62,63^ have found that earthworms and other soil fauna readily use such litter, high quality litter also decomposes rapidly without fauna and this masks faunal effects.

As shown by Frouz et al.^30^, the loss of organic matter in fauna-accessible litterbags may not equal mineralization, even in a majority of cases. Most litter consumed by soil fauna is transformed into faeces, which are incorporated into soil and can be decomposed by soil microorganisms^29^. The litter ingested by soil fauna and subjected to bioturbation follows a different path of decomposition than litter decomposing on the soil surface^64^. Bioturbation or ecosystem engineering by soil fauna alters the environment in which microorganisms integrate organic matter with mineral particles^65^. The bioturbation not only alters short-term decomposition but also alters microbial community composition and therefore exerts long-term effects on organic matter decomposition and sequestration in soil^66^. Litter processed by soil fauna follows a different trajectory of decomposition and transformation than that not consumed by soil fauna, an effect which confers greater ecological importance on the amount of litter consumed and processed through bioturbation.

Earthworms and other litter-feeding macrofauna dominate litter consumption in temperate climate regions, while termites dominate consumption in the tropics. This carries two important implications. First, both earthworms and other macrofauna prefer litter with a relatively high N content (i.e., litter with a low C:N ratio), while some termites feed on wood and other litter sources with high C:N ratios^3,5^. This suggests that soil fauna tend to consume low quality litter, i.e., litter with a high C:N ratios, to a greater degree in the tropics than in temperate regions. Second, termites accumulate food in their nests, which become hotspots of nutrient and energy transformation^61,67^. Although the faeces and tunnels of non-colonial, solitary fauna also represent hotspots, these are likely to be more evenly distributed in space than termite nests. Although difficult to measure, similar effects may apply to bioturbation. Earthworms, ants, and termites represent the most important bioturbators^3,5^.

Our results show that annual belowground herbivory accounts for about 4% of fine root biomass. In aboveground systems herbivores consume about 5 and 10% of NPP in forest and grassland ecosystem respectively^68^. Fine root biomass does not represent the entirety of belowground production and turnover rate likely varies between biomes, but we can expect that percentage of belowground NPP consumed by belowground herbivores remains lower than NPP consumed aboveground^68^. Our estimates found fine root biomass consumed by belowground herbivores being highest in boreal and temperate forests and lowest in Mediterranean biomes and deserts. We estimated that the quantity of plant material consumed by belowground herbivores (i.e., soil fauna that consume living plant material) was an order of magnitude lower than the quantity of plant material consumed by litter feeders (which consume dead plant material). These estimates also resemble previously published estimates^53^. The belowground herbivory appears higher in forests than in grasslands.

Many factors in the approach used here may lead to over- or underestimation of actual litter consumption and herbivory. We estimated annual consumption as daily consumption multiplied by number of days. However, fauna may become inactive for part of the year due to temperature or drought^69–71^. We used temperature corrections^72^ to correct for temperature effects across biomes. However, temperature effects depend on many other factors such as snow depth which, among other effects, may influence soil temperature in winter. Even in cold biomes snow cover can substantially increase winter activity of soil fauna^73^. The lack of consistent soil moisture corrections may affect estimates in arid biomes such as deserts or savannas. Laboratory mesocosms typically used in empirical studies provide fauna with food more readily than field environments such that experiments may overestimate the quantity of litter consumed by fauna.

Our quantitative methods interpreted average consumption of leaf litter that differed in terms of quality across biomes. Previous research has often interpreted litter of varying quality (including broadleaf, conifer, herbs, and grasses) with uniform estimates. Variation in litter may contribute to variation in consumption rates. Laboratory experiments also often measure consumption during mature developmental stages of consumers because these organisms are larger, more robust, and easier to handle than the same species in less mature stages. Mature consumers consume more food per capita than they do younger life stages, and this facilitates measurement of consumption. Frouz et al.^74^ showed that during ontogenetic development, food consumption per unit of biomass correlates negatively with animal size, i.e., immature individuals consume more food per unit of body mass than mature individuals. Failure to account for high food consumption rates per unit body mass during immature stages may therefore underestimate overall quantity of litter consumed.

Few studies have performed rigorous evaluation of the overall effect of these factors on annual litter consumption by soil fauna. To illustrate this, we analyzed monthly estimates of larval biomass and consumption rates (including temperature corrections) from Frouz et al.^75,76^ (Supplementary Fig. S1) to derive an estimate of annual litter consumption by March fly larval populations (*Penthetria holosericea*) as 134 g m^−2^ (dry weight). Omitting temperature corrections, using average consumption rates across all instars, and using average annual biomass however gives 114 g m^−2^. Using mean annual biomass and litter consumption by final instar (the approach often used in litter consumption studies) gives annual litter consumption of only 40 g m^−2^ (Supplementary Fig. S1). These particular studies suggest that using consumption based on larger and older life stages can substantially underestimates overal fauna consumtion. Given the temperature and moisture variation effects described above, additional effects may lurk in data from other biomes besides temperate forests. In the above example, simplified estimates all underestimate consumption compared to more detailed estimates, but this effect may not be generalizable to other studies. Future research can systematically characterize sources and impacts of errors.

### Distribution of soil fauna functional groups across biomes

In agreement with Petersen and Luxton^69^ and Fierer et al.^12^, the present study found that the total soil fauna biomass and functional group biomass differed among global biomes. Previous reports have found the highest levels of aboveground plant and animal biomass in tropical biomes with decreasing trends towards higher latitudes^77–80^. The present study and that of Petersen and Luxton^69^ have instead found the highest soil faunal belowground biomass in temperate grassland, followed by boreal and temperate forests. The high biomass of soil fauna in temperate grasslands apparently arises from the large input of litter (i.e., food for soil fauna) with low C:N and lignin:N ratios. It is well known that substantial production of high-quality litter supports high numbers of earthworms and other bioturbators^3,57^ that increase the humus layer. Temperate grasslands typically have a thick A horizon in which humus provides a habitat for other soil fauna that contribute to litter decomposition^81,82^.

Our review indicated that termite biomass was higher in tropical grasslands, Mediterranean ecosystems, and deserts than in other biomes. We suggest that the higher biomass of termites in the warmer biomes depends mostly on climatic factors (higher temperatures) and litter chemistry (higher C:N ratios)^83,84^. Our finding is consistent with those of Blanchart et al.^84^ who showed that termites represented the largest proportion of faunal biomass in tropical grasslands. These findings were also supported by our NMDS analyses, which indicated that temperature, precipitation, and mean annual litterfall were the main determinants of faunal biomass in tropical regions. Both the current and previous studies found positive correlations between biomass of earthworms in temperate grasslands and the quantity of available nutrients in the A horizon^66,85,86^. Our results also showed that omnivore and earthworm biomasses in temperate biomes correlated positively with soil N content. Furthermore, our review identified large biomass of fungal feeders and predators in colder biomes. We hypothesize that larger biomass of microbial feeders may result from bottom-up effects of microbial biomass on microbial feeders and of microbial feeders on predators^87^.

Our study showed that the biomass of some soil fauna functional groups correlated with climatic factors as well as with litter quality and that the effects of climatic factors and litter quality varied across biomes. For example, soil fauna biomasses were most closely related to the C:N ratio of litter in tundra, taiga, and Mediterranean biomes but with temperature, precipitation, and NPP in tropical biomes. We suggest that the significant correlations between soil fauna biomass and climatic variables and litter quality reflect differences in vegetation type and soil microbial patterns at a global scale^88^. In agreement with Bardgett and van der Putten^1^, we also suggest that climatic factors can affect soil fauna biomass directly^89^ but also indirectly by affecting litter quality^28,56^. Portela et al.^89^ for example reported increased abundance of soil fauna during a rainy season with sufficient precipitation. In addition, Prieto et al.^90^ found that the quality of leaf litter produced by shrubs decreased in plots that were experimentally warmed. We suggest that coupled effects of climate and litter quality change the physiology and growth of soil fauna. By changing functional responses and biotic interactions, changes in physiology and growth substantially affect the diversity and community structure of soil organisms^1,28,91^.

## Conclusions

Our quantitative review revealed that the biomass of soil fauna functional groups varied across biomes that differ in terms of climate and litter quality. Whereas previous studies report the highest aboveground plant and animal biomass in tropical biomes with decreasing biomass towards higher latitudes, the present study found the highest belowground soil fauna functional group biomass in temperate grasslands with decreasing biomass as follows: boreal forest > temperate forest > tropical grassland > tundra > tropical forest > Mediterranean ecosystems > desert and semidesert biomes. Tropical grasslands, deserts, and Mediterranean ecosystems were dominated by termites. Temperate grasslands were dominated by omnivores, and temperate forests were dominated by earthworms. The biomass of soil fauna was lower in arid and nutrient-poor biomes than in humid and nutrient-rich biomes. Across biomes, soil fauna were estimated to consume approximately 50% of the annual litterfall. The estimated percentage of litter consumption among biomes ranged from 14.9% in deserts to 100.4% in temperate grassland.

## Materials and methods

### Distribution and biomass of soil fauna functional groups

To investigate soil fauna functional group biomass across biomes, we searched for journal articles published before December 31, 2019 using the Web of Science, Google Scholar, and Scopus using the search terms “Polar regions OR Tundra, OR Cold steppe OR Boreal forest OR Taiga OR Temperate forest OR Temperate grassland OR Steppe OR Shrubland OR Mediterranean vegetation OR Desert OR Semi-desert OR Dryland OR Arid OR Tropical grassland OR Savannah OR Tropical forest AND soil fauna OR Soil animals” in English. We used data from observational studies, control sites in litterbag experiments (i.e., sites or plots without litterbags), and control or untreated sites or plots in manipulative field experiments. We considered studies of all soil fauna which used wet or dry extraction and hand sorting (except some dry-desert sites which mostly used pitfall traps due to the absence of soil organic layers) for sampling of soil fauna. We included data from topsoil (0–10 cm). We did not use data from laboratory or field studies that used litterbag treatments or other kinds of treatments. If results from the same study sites and the same sampling year were reported in different articles, only one article was included in our database.

A total of 70 studies with > 1000 observations matched the selection criteria described above. The biomass within each study was calculated for various functional groups of soil fauna (Table S1). The average biomass of individual faunal groups from different studies was calculated for each biome (Supplementary Table S1). If a study provided data only on faunal population density, we estimated the total biomass from the average dry weight of one individual based on previous reports^36,69^. Following published studies^92–95^ we distinguished four main functional groups of soil fauna: herbivores, omnivores, predators, and saprotrophs. Saprotrophs represent the most abundant group of soil fauna. This group consists of four sub-groups: bacterial feeders, fungal feeders, and litter-feeding macro- and mesofauna. We decided to separate specific taxonomic groups because of their different functions in soil ecosystems. We distinguished groups having sufficiently uniform ecology such that they will likely exhibit similar within group features in terms of foraging pattern and litter processing. Omnivores were divided into ants and other omnivores because ants are central place foragers. Similarly, we categorized litter feeding macrofauna as termites, earthworms, and other litter feeding macrofauna to emphasize the specific role of earthworms in soil processes and the fact that termites are central place foragers. To make our categories clear we use only the last (most detailed category) and skip others. Table 1 and Supplementary Table S1 list group names in bold along with their position.

### Annual litterfall, fine root biomass, potential litter consumption, substrate quality, and climate data

Climatic data (temperature and precipitation), net primary production (NPP), soil chemistry (C:N and N), and litter quality (N content) were obtained from previously published literature sources (Table 3). Fine root biomass was used from a database established by Jackson et al.^96,97^ that includes around 250 studies published from 1950 to 1995. To assess mean annual litterfall and potential litter consumption by soil faunal groups across biomes, journal articles published before December 31, 2019 were searched using the Web of Science, Google Scholar, and Scopus with the search terms “Polar regions OR Tundra, OR Cold steppe OR Boreal forest OR Taiga OR Temperate forest OR Temperate grassland OR Steppe OR Shrubland OR Mediterranean vegetation OR Desert OR Semi-desert OR Dryland OR Arid OR Tropical grassland OR Savannah OR Tropical forest AND annual litterfall AND/OR litter consumption” in English. As previously indicated for biomass data, if litterfall or litter consumption data from the same study sites and the same sampling year were reported in different studies, only one study was included in our database. In total, we found 24 studies based on 692 observations for mean annual litterfall, 28 studies based on 183 observations for mean litter stock (Supplementary Table S2), and 44 studies based on 200 observations for consumption of plant litter and other plant material by soil invertebrate faunal groups (Supplementary Table S3). Litter data included leaves, needles, and also fine branches. Fine root biomass referred to biomass of roots ≤ 2 mm in diameter. We calculated turnover rate (ratio of mean annual litterfall and mean litter stock) and turnover time (ratio of mean litter stock and mean annual litterfall) for each biome.

**Table 3:** Sources of data for net primary production (NPP), mean annual temperature (MAT), mean annual precipitation (MAP), N content of soil, N content of litter, and the soil C:N ratio in biomes.

Biome	NPP (Gt C yr^-1^)	MAT (°C)	MAP (mm)	N soil (mg kg^-1^)	N litter (mg kg^-1^)	CN ratio (soil)	References
Tundra and cold steppe	1.3 ± 1.1	− 0.2 ± 0.1	483 ± 219	0.86 ± 0.23	1.1 ± 0.9	72 ± 64	^77,82,99–101^
Boreal forest	6.6 ± 2.5	0.3 ± 0.09	643 ± 332	0.96 ± 0.45	5.1 ± 2.2	68 ± 42	^77,79,99,80–104^
Temperate forest	9.3 ± 3.8	5.8 ± 1.1	735 ± 344	1.02 ± 0.55	7.2 ± 1.9	72 ± 23	^77,79,99,101–105^
Temperate grassland	7.5 ± 2.2	6.8 ± 3.4	550 ± 267	1.37 ± 0.62	4.8 ± 2.4	47 ± 28	^79,99,101,102,104,106^
Mediterranean vegetation	7.2 ± 1.9	14.7 ± 6	1320 ± 550	0.86 ± 0.44	3.8 ± 1.5	72 ± 31	^79,99,101,102,107^
Desert and semidesert	3.5 ± 1.2	8.6 ± 3.9	74 ± 19	0.87 ± 0.43	0.7 ± 0.2	54 ± 22	^79,99,80,101,104^
Tropical grassland	14.9 ± 5.9	19.9 ± 7.2	1350 ± 560	0.97 ± 0.27	9.2 ± 4.8	54 ± 25	^78,79,99,80,101,104^
Tropical forest	22.5 ± 12.4	21.4 ± 11.9	1590 ± 698	0.95 ± 0.38	10.1 ± 5.1	56 ± 21	^79,81,99,80,102–104,108,109^

Holland et al.^99^ includes data from 685 original literature sources dating from 1827 to 1997.The N litter was obtained from fallen litter while soil related data were obtained from topsoil (0–10 cm).

In some cases, faunal consumption was measured for a short period that enabled the estimation of daily consumption. In these cases, annual consumption was calculated by multiplying daily consumption by 365. For each feeding group (including belowground herbivores), we then calculated mean annual litter consumption (root consumption in case of belowground herbivores), and this was used across all biomes. We included both field and laboratory studies that measured faunal consumption if they enabled the estimation of faunal consumption in terms of dry mass consumed per mg of animal per day. Except for termites, most of the available data were derived from temperate biomes. We then multiplied a summary of the biomasses of saprotrophs, earthworms, termites, and belowground herbivores in each biome by mean annual consumption to estimate faunal consumption in terms of dry mass consumed per mg of animal per year. To adjust for higher or lower invertebrate activity in warmer or colder biomes, we used the mean temperature for temperate biomes as a baseline and corrected the consumption in other biomes according to the mean annual temperature in that a particular biome. This calculation was based on a formula describing the universal dependence of metabolism on temperature^72^.

### Statistical analyses

Non-metric multidimensional (NMDS) scaling was used to investigate the relationships between the distribution of soil fauna functional groups, climatic variables, and litter quality. Pearson’s correlation analysis was used to determine how consumption of litter or live, belowground plant tissue by soil fauna was related to climatic factors and litter quality for each biome. Bonferroni corrections were used to adjust the p value of multiple correlations. Partial least squares path modeling (PLS-PM) was used to analyze the effects of climate (MAT, MAP), soil (soil N and C:N ratio), and productivity (NPP, N (litter), and mean annual litterfall) on soil fauna functional group biomass associated with litter transformation (earthworms, termites, saprotrophs, herbivores, litter feeding mesofauna and macrofauna). The PLS-PM was calculated using the ‘plspm’ package^98^.

## Supplementary Information

Supplementary Information.

## Data Availability

Full data are provided in Supporting Information Tables S1–S3. Other data will be provided by corresponding author, Jan Frouz upon request.
