# Supplementary material for: Global distribution of soil fauna functional groups and their estimated litter consumption across biomes
Source: Sci Rep. 2022 Oct 17;12:17362. doi: 10.1038/s41598-022-21563-z (PMC9576680; doi:10.1038/s41598-022-21563-z)
Supplement: Supplementary file 1 — Supplementary Information. [file 41598_2022_21563_MOESM1_ESM.docx]

**Global distribution of soil fauna functional groups and their estimated litter consumption across biomes**

Petr Heděnec ^1,2^, Juan Jose Jiménez ^3^, Jabbar Moradi ^1,11^, Xavier Domene ^4,5^, Davorka Hackenberger ^6^, Sebastien Barot ^7^, Aline Frossard ^8^, Lidia Oktaba ^9^, Juliane Filser ^10^, Pavel Kindlmann^11^, and Jan Frouz ^1,11*^

^1^ Institute of Soil Biology and SoWa, Biology Centre ACR, Na Sádkách 7, České Budějovice, 37005 Czech Republic

^2^ Institute of Tropical Biodiversity and Sustainable Development, University Malaysia Terengganu, 21030, Kuala Nerus, Terengganu, Malaysia

^3^ Pyrenean Institute of Ecology, IPE-CSIC, Avda, Ntra, Sra, de la Victoria, 16, Jaca, 22700, Huesca, Spain

^4^ CREAF, E08193 Cerdanyola del Vallès 08193, Spain

^5^ Universitat Autònoma de Barcelona, Cerdanyola del Vallès 08193, Spain

^6^ Josip Juraj Strossmayer University of Osijek, Department of Biology, Cara Hadrijana 8/A, HR-31000 Osijek, Croatia

^7^ IEES-Paris (CNRS, UPMC, IRD, INRA, UPEC), UPMC 4 place Jussieu, 75252 Paris Cedex 05, France

^8^ Swiss Federal Research Institute WSL, Zürcherstrasse 111, 8903 Birmensdorf, Switzerland

^9^ Soil Science Department, Agriculture Institute, Warsaw University of Life Sciences-SGGW, Nowoursynowska 166, 02-787 Warsaw, Poland

^10^ Department of General and Theoretical Ecology, University of Bremen, FB 02, UFT – Centre for Environmental Research and Sustainable Technology, Leobener Straße 6, 28359, Bremen, Germany

^11^Institte for Environmental Studies, Faculty of Science, Charles University, Benátská 2, Praha 12800 Czech Republic

***Corresponding author: Jan Frouz**

**Email address:** [**frouz@natur.cuni.cz**](mailto:frouz@natur.cuni.cz)

**Supplementary methods**

**Method S1:**

**Why we assume that the amount of litter consumed by soil fauna has to be higher than the net difference between fauna-accessible and fauna-inaccessible litter bags**

Net fauna effect on decomposition is usually calculated as the net difference (ND) between the weight of contents of fauna-accessible and fauna-inaccessible enclosures, typically litterbags, at the instant of fauna effect evaluation. Let us assume we have two litterbags, both filled by the same amount of the same litter, Mi, where one is accessible to soil fauna and the other is not. Then some part of the litter (between 0 and 100 %) in the fauna-accessible bag is consumed by the fauna and hence affected by it, while the rest of litter in this litterbag is unaffected by the fauna. In the fauna non-accessible litter bag no such fauna-affected part exists. However consumed part of litter do not disappear, part of it is assimilated by soil fauna and part returned in to litterbag in form of feces.

Then we can write the net difference, ND, between the contents of fauna-accessible and fauna-inaccessible bags as follows

ND = (Mi – DL)-(p*((Mi – DLF)+(1-p)*(Mi-DL))), (1)

where Mi is the initial mass of litter in each of the litterbags,

DL is decomposed mass of litter at the time of fauna effect evaluation (this includes all mass loss of litter from the bag due to mineralization leaching. etc.) at the fauna-inaccessible bag, calculated for the initial mass, Mi.

DLF is decomposed mass of litter affected by fauna at the time of fauna effect evaluation (this includes all mass loss of fauna-consumed litter which may happed due to fauna assimilation, mineralization of organic matter in remaining feces, leaching from feces, etc.) , calculated for the initial mass, Mi (in reality, the decomposed mass is therefore p*DLF, and p*Mi is eaten).

p is proportion of original litter eaten by the fauna in the fauna-inaccessible litter bag

Formally, we can write

(Mi – DL) = p*(Mi – DL) + ((1-p)* (Mi – DL)) (2)

and substitution of (2) into (1) gives

ND = p*(Mi – DL)+ ((1-p)* (Mi – DL)) - ((p*(Mi – DLF)+ ((1-p)*(Mi-DL)). (3)

Using simple algebra, this can be simplified to

ND = p*(DLF -DL). (4)

Thus, for p = 0, it is ND = 0, as expected (no fauna – no difference between the bags). When fauna is present in the first bag, then p > 0 and ND is positive or negative, depending on whether DLF > DL or vice versa. Given DLF and DL, it holds: the larger the p, the larger the absolute value of ND.

If the amount of litter consumed by the soil fauna were equal to the net difference between the contents of fauna-accessible and fauna-inaccessible litter bags at the instant of fauna effect evaluation, then it would have to be

ND = p*Mi (5)

(the amount of litter consumed by soil fauna is on the right-hand side, the net difference between the contents of fauna-accessible and fauna non-accessible litter bags is on the left-hand side). Comparing (4) with (5) gives

Mi = DLF-DL. (6)

Verbally: In order for (6) to hold, the difference between: (i) the amount of mass decomposed in the fauna-accessible bag until the instant of fauna effect evaluation, calculated for the initial mass, Mi (DLF) and (ii) the amount of mass decomposed in the fauna-inaccessible bag until the instant of fauna effect evaluation (DL) must be equal to the litter biomass in each of the bags at the beginning, Mi. As clearly DL > 0 (at least some decomposition occurs in the fauna-inaccessible bag) and DLF < Mi (the fauna is unable to feed and/or cause decomposition of more than the total contents of the fauna-accessible bag, Mi), equality in (6) can only occur when DLF = Mi (fauna eats up the whole amount of the bag, which would be either completely assimilated i.e. no feces are produced or feces of fauna would completely decompose until the instant of fauna effect evaluation) and simultaneously DL = 0 (there is no decomposition in the fauna non-accessible bag). The situation when both of these conditions are met at the same time is an extreme, biologically unrealistic case. Therefore, it must hold (except of the just mentioned unrealistic case) in (6):

Mi > DLF-DL (8)

or else

p*Mi > p*(DLF-DL) (9)

And using (4) and the knowledge that p*Mi is the amount of litter eaten by the fauna:

ND < p*Mi, (10)

i.e., **the net difference (ND) between the weight of contents of fauna-accessible and fauna-inaccessible bags (see (4) is smaller than the total amount of litter eaten by fauna until the instant of fauna effect evaluation, p*Mi.**

Let us illustrate this on an example (Fig. 1). The example is adjusted to fit the equations above (namely equation 8 and 10) but based on real world data from Frouz and Simek^1^ . Fig. 1 shows mass loss of litter in situation, when all litter in fauna-accessible bag was consumed (p=1), converted to feces which stay in the bag (Mi is 100% for both fauna-affected as well as fauna-unaffected litter). Part of the liter eaten by fauna was assimilated by fauna, which is responsible for raid mass loss early after the start of experiment (note that the x axis is logarithmic). However most of the mass of litter eaten by fauna was turned in fauna feces. Then the fauna disappeared from the bag and both intact litter as well as feces of fauna (i.e. leftover of litter processed by fauna) decompose, which is manifested by the decrease of the mass over time in both types of bags. At the beginning, the fauna-unaffected litter had higher remaining mass than the fauna-affected litter (feces), later this difference has disappeared. This is reflected by the difference between mass of both bags (ND in equations above), which increase early after the start and then gradually decrease. However, even the maximal value of ND reaches only about 20% of the amount of litter consumed by soil fauna (i.e. ND max reaches 20% of Mi in equations above). This means that when we use the difference in weights of the remaining litter between fauna-affected bag and fauna-unaffected bags as a proxy for the amount of litter consumed by soil fauna, this would cause at least a 5 fold underestimate. The level of this underestimation will increase substantially with time: in bags older than half a year, ND would be 0 or lower, but the amount of litter consumed by fauna would still be the same (Fig. 1).


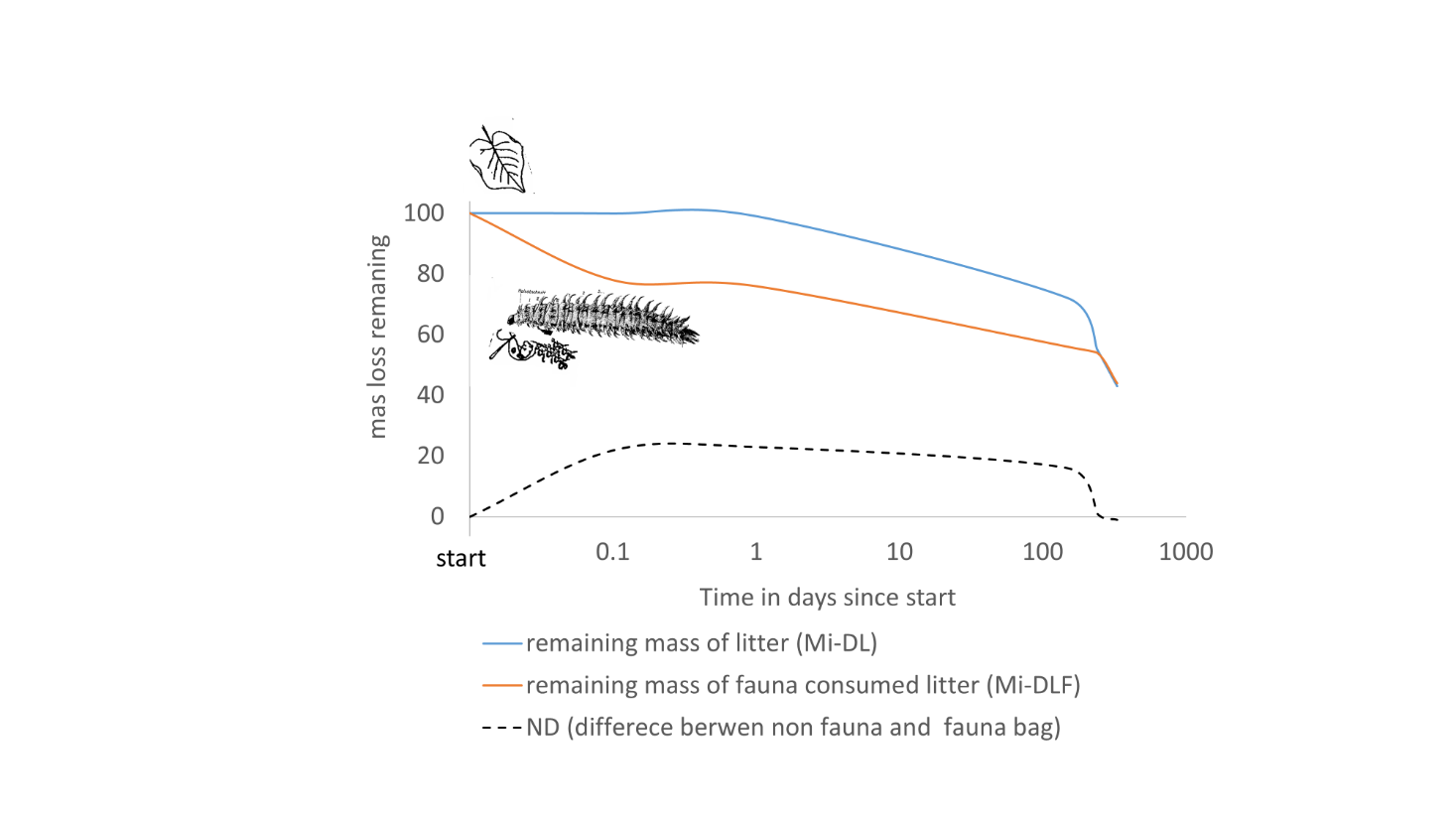


Fig. 1. Remaining mass of litter in litterbag with no fauna access, (Mi-DL) and remaining mass of fauna consumed litter (Mi-DLF) and difference between those two (ND).

As a matter of fact, in studies that compare difference in mass loss between litter and feces^1,2^ this difference was rather in percent or tens of percent than order of magnitude so very likely total amount of litter consumed by fauna should be several fold higher than net difference that appear from enclosure studies.

**References:**

1. ^1^Frouz, J., Šimek, M.. Short term and long term effects of bibionid (Diptera: Bibionidae) larvae feeding on microbial respiration and alder litter decomposition. *European Journal of Soil Biology*, **45**: 192-197. (2009)
2. ^2^Frouz, J., Roubíčková, A., Heděnec, P., Tajovský, K. Do soil fauna really hasten litter decomposition? A meta-analysis of enclosure studies. *European Journal of* *Soil Biology*, **68**: 18-24 (2015).

**Supplementary figure 1**


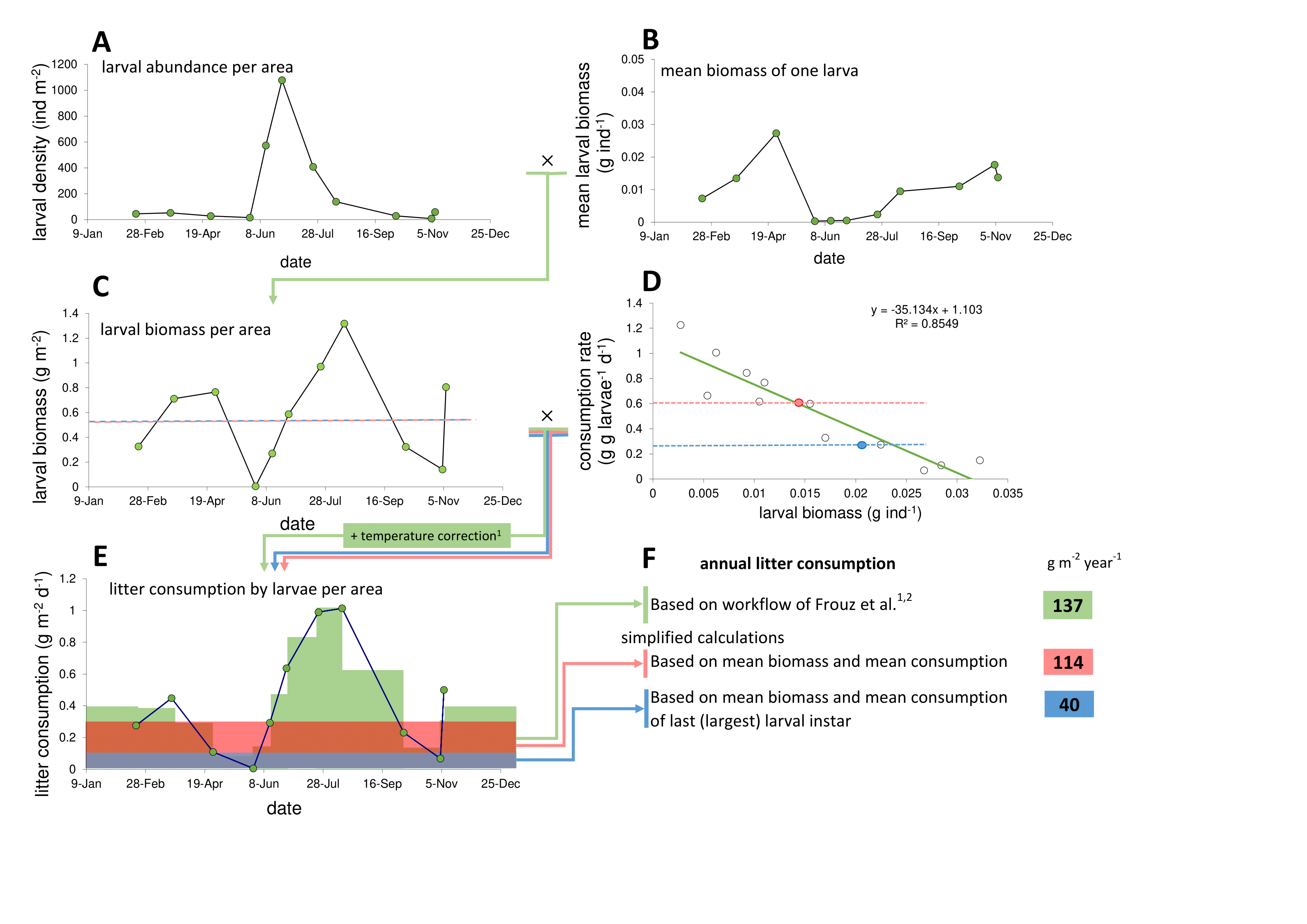


Figure S1: Comparison of the workflows for calculating the annual litter fauna consumption based on detailed data^1,2^ (in green) and based on simplified calculations (in red and blue). All weights are given as dry weights, $\times$ denotes multiplication.

**Workflow of Frouz et al.^1,2^ calculation**

1) larval density (Fig. S1A) and larval size was measured in individual sampling occasions

2) a subset of larvae were weighed and their individual biomass and size were used to develop

an allometric equation

3) larval biomass per individual of the remaining larva was estimated from larval size using

the allometric equation and a mean biomass value was calculated for each sampling occasion (Fig. S1B)

3) larval biomass per area (Fig. S1C) was calculated by multiplying larval density and mean biomass for each sampling occasion.

4) consumption rate (Fig. S1D) was measured for full range of biomass values recorded

5) litter consumption rate in each sampling period (Fig. S1E) was calculated using biomass per area

and biomass specific consumption rate and temperature correction based on Frouz et al.^1^

6) consumption in individual sampling periods was integrated into an annual value (Fig. S1F)

**Simplified calculations**

Simplified calculations use no temperature corrections and assume no seasonal changes in biomass.

They are based on multiplication of mean annual biomass and mean consumption rate (Fig. S1 in red; the same approach was used in this quantitative review) or on mean annual biomass and consumption of last larval instar (Fig. S1 in blue) assuming the largest animals are often used in consumption studies.

**References:**

1. Frouz, J., Šustr, V. & Kalčík, J. Energetic budget of three species of bibionid larvae. *Contributions to Soil Zoology in Central Europe I. ISB AS CR, České Budějovice* 15–18 (2005).

2. Frouz, J., Jedlička, P., Šimáčková, H. & Lhotáková, Z. The life cycle, population dynamics, and contribution to litter decomposition of *Penthetria holosericea* (Diptera: Bibionidae) in an alder forest. *Eur. J. Soil Biol.* **71**, 21–27 (2015).

**Supplementary tables:**

Table S1: Mean biomass (±SE) of functional groups of soil fauna across global biomes. n – number of observations.

| Functional groups* | Biomes | Biomass (mg m^2^ dw) | n | Reference |
| --- | --- | --- | --- | --- |
| Bacterial feeders | Tundra and cold steppe | 126.79±114.7 | 6 | ^1–4^ |
| Earthworms | Tundra and cold steppe | 85.22±81.6 | 4 | ^2–7^ |
| Fungal feeders | Tundra and cold steppe | 413.37±341.1 | 4 | ^1,2,4,5^ |
| Herbivores | Tundra and cold steppe | 44 | 1 | ^1^ |
| Litter feeders:macrofauna | Tundra and cold steppe | 318 | 1 | ^1^ |
| Litter feeders:mesofauna | Tundra and cold steppe | 10 | 1 | ^2^ |
| Omnivores | Tundra and cold steppe | 51.95±49.7 | 11 | ^2–5^ |
| Predators | Tundra and cold steppe | 121.86±77.1 | 9 | ^1,2,4^ |
| Saprotrophs | Tundra and cold steppe | 695.66±585.8 | 4 | ^1,2^ |
| Bacterial feeders | Boreal forest | 160.49±159.8 | 6 | ^1,4,8^ |
| Earthworms | Boreal forest | 147.46±128.3 | 5 | ^1,4–7^ |
| Fungal feeders | Boreal forest | 976.95±372.2 | 17 | ^1,5,9–11^ |
| Herbivores | Boreal forest | 194.56±176.8 | 10 | ^8,9,11^ |
| Litter feeders:macrofauna | Boreal forest | 290.42±130.3 | 9 | ^1,8,11^ |
| Litter feeders:mesofauna | Boreal forest | 215.20±124.0 | 4 | ^1,10,11^ |
| Omnivores | Boreal forest | 224.22±100.5 | 19 | ^4,5,8–11^ |
| Predators | Boreal forest | 1745.06±1609 | 26 | ^1,4,8–11^ |
| Saprotrophs | Boreal forest | 323.49±158.5 | 11 | ^4,10–13^ |
| Ants | Temperate forest | 11.75±4.6 | 5 | ^2,7,14,15^ |
| Bacterial feeders | Temperate forest | 369.75±205.2 | 23 | ^1,2,4,15–19^ |
| Earthworms | Temperate forest | 1628.03±1514.7 | 9 | ^2,4–6,16,19–21^ |
| Fungal feeders | Temperate forest | 79.62±34.7 | 31 | ^2,5,14–19,22–27^ |
| Herbivores | Temperate forest | 190.53±92.2 | 11 | ^4,15–17,19,21,26^ |
| Litter feeders:macrofauna | Temperate forest | 46.40±26.2 | 11 | ^2,14–19,22,26^ |
| Litter feeders:mesofauna | Temperate forest | 72.36±61.5 | 18 | ^2,17,18,23,24,27,28^ |
| Omnivores | Temperate forest | 280.68±148.8 | 29 | ^2,4,17,18,22–27,29^ |
| Predators | Temperate forest | 151.93±83.0 | 39 | ^2,4,15,16,18,19,23,24,26,27^ |
| Saprotrophs | Temperate forest | 362.54±191 | 23 | ^2,4,13–16,19,22,26^ |
| Termites | Temperate forest | 5±2.5 | 2 | ^30,31^ |
| Ants | Temperate grassland | 33.48±33.3 | 3 | ^2,7,14,32^ |
| Bacterial feeders | Temperate grassland | 60.08±40.5 | 12 | ^2,4,33–36^ |
| Earthworms | Temperate grassland | 1821.56±1557.9 | 16 | ^2,4–7,20,32,34,35,37–42^ |
| Fungal feeders | Temperate grassland | 62.23±32.9 | 25 | ^2,5,14,27,33–36,41–45^ |
| Herbivores | Temperate grassland | 41.67±24.6 | 6 | ^4,33,35,42^ |
| Litter feeders:macrofauna | Temperate grassland | 201.25±199.7 | 7 | ^2,14,27,39,42^ |
| Litter feeders:mesofauna | Temperate grassland | 35.74±17.2 | 10 | ^2,27,39,43–45^ |
| Omnivores | Temperate grassland | 4930.65±4804 | 27 | ^2,4,14,20,34–36,39,41–48^ |
| Predators | Temperate grassland | 56.73±33.7 | 28 | ^2,4,33,35,36,39,41,42,44^ |
| Saprotrophs | Temperate grassland | 51.75±38.5 | 15 | ^2,4,27,32,33,35,39,41,42^ |
| Termites | Temperate grassland | 200±98.9 | 4 | ^7,30–32^ |
| Ants | Mediterranean vegetation | 1.26±1.2 | 19 | ^49,50^ |
| Earthworms | Mediterranean vegetation | 157.50±157.5 | 3 | ^6,7,50,51^ |
| Fungal feeders | Mediterranean vegetation | 15.02±15.0 | 6 | ^35,50,52^ |
| Herbivores | Mediterranean vegetation | 0.37±0.36 | 47 | ^49,50^ |
| Litter feeders:macrofauna | Mediterranean vegetation | 0.09±0.09 | 27 | ^49,50^ |
| Litter feeders:mesofauna | Mediterranean vegetation | 30.62±30.6 | 4 | ^50,52^ |
| Omnivores | Mediterranean vegetation | 36.05±36.0 | 9 | ^50,52^ |
| Predators | Mediterranean vegetation | 6.82±6.7 | 63 | ^49,50,52^ |
| Saprotrophs | Mediterranean vegetation | 0.45±0.1 | 14 | ^49,50^ |
| Termites | Mediterranean vegetation | 323.33±323.3 | 6 | ^7,30,31,49,53^ |
| Ants | Desert and semideserts | 3.03±1.5 | 4 | ^7,54^ |
| Bacterial feeders | Desert and semideserts | 0.39 | 1 | ^54^ |
| Fungal feeders | Desert and semideserts | 0.14±0.1 | 2 | ^5,54^ |
| Herbivores | Desert and semideserts | 4.63±2.4 | 7 | ^54^ |
| Omnivores | Desert and semideserts | 10.15±7.9 | 23 | ^5,54^ |
| Predators | Desert and semideserts | 15.72±15.2 | 18 | ^4,54^ |
| Saprotrophs | Desert and semideserts | 2.66±1.8 | 2 | ^51,54^ |
| Termites | Desert and semideserts | 300±289 | 4 | ^30,31,53,55^ |
| Ants | Tropical grassland | 150.18±149.8 | 3 | ^2,7,56^ |
| Bacterial feeders | Tropical grassland | 105.16±104.8 | 21 | ^2,57^ |
| Earthworms | Tropical grassland | 28.37±28.3 | 20 | ^2,6,7,56–60^ |
| Fungal feeders | Tropical grassland | 5.07±4.9 | 9 | ^2,57^ |
| Herbivores | Tropical grassland | 5.23±4.8 | 11 | ^2,57^ |
| Litter feeders:macrofauna | Tropical grassland | 10.00 | 1 | ^2^ |
| Litter feeders:mesofauna | Tropical grassland | 50.00 | 1 | ^2^ |
| Omnivores | Tropical grassland | 65.05±64.9 | 11 | ^2,57^ |
| Predators | Tropical grassland | 18.42±18.3 | 8 | ^2,56,57^ |
| Saprotrophs | Tropical grassland | 40.00±36.0 | 2 | ^2,61^ |
| Termites | Tropical grassland | 2247.10±1927.4 | 6 | ^2,7,30,56,58^ |
| Ants | Tropical forest | 4.52±3.7 | 9 | ^2,7,62–67^ |
| Bacterial feeders | Tropical forest | 0.92±0.9 | 6 | ^4,64,66^ |
| Earthworms | Tropical forest | 39.84±37.5 | 10 | ^2,4–6,62–64,66^ |
| Fungal feeders | Tropical forest | 5.63±4.8 | 14 | ^2,5,67,68^ |
| Herbivores | Tropical forest | 14.24±12.9 | 38 | ^2,4,61–69^ |
| Litter feeders:macrofauna | Tropical forest | 162±5 | 22 | ^2,62–70^ |
| Litter feeders:mesofauna | Tropical forest | 16 | 1 | ^70^ |
| Omnivores | Tropical forest | 212.60±194.5 | 46 | ^2,4,61–69^ |
| Predators | Tropical forest | 352.06±348.4 | 47 | ^2,4,61–69^ |
| Saprotrophs | Tropical forest | 38.46±34.2 | 23 | ^2,4,61–65,67–69^ |
| Termites | Tropical forest | 809.25±523.0 | 14 | ^2,7,30,61–66,68,69^ |

*Examples of functional groups of soil fauna:

Bacterial feeders: Protists, bacterivorous nematodes, dipteran larvae, rotifers, tardigrades.

Fungal feeders: Oribatid mites, collembolans, pauropods, proturans, fungivorous nematodes.

Herbivores: Gastropods, hemipterans, homopterans, herbivorous nematodes.

Litter feeding macrofauna: Unidentified soil fauna larger than 2 mm.

Litter feeding macrofauna: Unidentified soil fauna smaller than 2 mm.

Omnivores: Blattodea, Dermaptera, myriapods *sensu lato,* omnivorous nematodes, insects *sensu lat.*

Predators: Spiders, centipedes, Mesostigmats, Diplura.

Saprotrophs: Saprotrophic nematodes, isopods, milipedes, enchytreids.

**References:**

1. Huhta, V. & Koskenniemi, A. Numbers, biomass and community respiration of soil invertebrates in spruce forests at two latitudes in Finland. *Anna. Zool. Fenn.* **12**, 164–182 (1975).

2. Petersen, H. & Luxton, M. A comparative analysis of soil fauna populations and their role in decomposition processes Author ( s ): Henning Petersen and Malcolm Luxton Source : Oikos , Vol . 39 , No . 3 , Quantitative Ecology of Microfungi and Animals in Soil a. **39**, (1982).

3. Simmons, B. L. *et al.* Terrestrial mesofauna in above- and below-ground habitats: Taylor valley, Antarctica. *Pol. Biol.* **32**, 1549–1558 (2009).

4. Little, C. The Terrestrial Invasion: an Ecophysiological Approach to the Origins of Land Animals. Cambridge University Press, Cambridge. (1990).

5. Fierer, N., Strickland, M. S., Liptzin, D., Bradford, M. A. & Cleveland, C. C. Global patterns in belowground communities. *Ecol. Lett.* **12**, 1238–1249 (2009).

6. Phillips, H. R. P. *et al.* Global distribution of earthworm diversity. *Science* **366**, 480–485 (2019).

7. Orgiazzi, A. *et al.* *Global Soil Biodiversity Atlas*. (2016).

8. Paquin, P. & Coderre, D. Changes in soil macroarthropod communities in relation to forest maturation through three successional stages in the Canadian boreal forest. *Oecologia* **112**, 104–111 (1997).

9. Gongalsky, K. B. *et al.* Do burned areas recover from inside? An experiment with soil fauna in a heterogeneous landscape. *Appl. Soil Ecol.* **59**, 73–86 (2012).

10. Kataja-aho, S., Fritze, H. & Haimi, J. Short-term responses of soil decomposer and plant communities to stump harvesting in boreal forests. *For. Ecol. Manag.* **262**, 379–388 (2011).

11. Salmon, S., Mantel, J., Frizzera, L. & Zanella, A. Changes in humus forms and soil animal communities in two developmental phases of Norway spruce on an acidic substrate. *For. Ecol. Manag.* **237**, 47–56 (2006).

12. Haimi, J. *et al.* Impacts of elevated CO_2_ and temperature on the soil fauna of boreal forests. *Appl. Soil Ecol.* **30**, 104–112 (2005).

13. Waldrop, M. P. *et al.* Bacterial and enchytraeid abundance accelerate soil carbon turnover along a lowland vegetation gradient in interior Alaska. *Soil Biol. Biochem.* **50**, 188–198 (2012).

14. Barbercheck, M. E., Neher, D. A., Anas, O., El-Allaf, S. M. & Weicht, T. R. Response of soil invertebrates to disturbance across three resource regions in North Carolina. *Env. Monitor Assess.* **152**, 283–298 (2009).

15. Persson, T. *et al.* trophic structure, biomass dynamics and carbon metabolism of soil organisms in a scots pine forest. *Structure and Function of Northern Coniferous Forests - An Ecosystem Study* **32**, 419–459 (1980).

16. Irmler, U. Changes in the faua and its contribution to mass loss and N release during leaf litter decoimposition in the wood deciduous forest. **118**, 105–118 (2000).

17. Jandl, R., Kopeszki, H., Bruckner, A. & Hager, H. Forest soil chemistry and mesofauna 20 years after an amelioration fertilization. *Restor. Ecol.* **11**, 239–246 (2003).

18. Maraun, M. *et al.* Indirect effects of carbon and nutrient amendments on the soil meso- and microfauna of a beechwood. *Biol Fert. Soils* **34**, 222–229 (2001).

19. Schaefer, M. & Schauermann, J. The soil fauna of beech forests: comparison between a mull and a moder soil. *Pedobiologia* **34**, 299–314 (1990).

20. Melody, C. & Schmidt, O. Northward range extension of an endemic soil decomposer with a distinct trophic position. *Biol. Lett.* **8**, 956–959 (2012).

21. Scheu, S. Effects of earthworms on plant growth: patterns and perspectives. *Pedobiologia* **47**, 846–856 (2003).

22. Battigelli, J. P., Berch, S. M. & Marshall, V. G. Soil fauna communities in two distinct but adjacent forest types on northern Vancouver Island, British Columbia. *Can. J. For. Res.* **24**, 1557–1566 (1994).

23. Eisenhauer, N., Partsch, S., Parkinson, D. & Scheu, S. Invasion of a deciduous forest by earthworms: Changes in soil chemistry, microflora, microarthropods and vegetation. *Soil Biol. Biochem.* **39**, 1099–1110 (2007).

24. Gonzalez-Polo, M., Fernández-Souto, A. & Austin, A. T. Coarse woody debris stimulates soil enzymatic activity and litter decomposition in an old-growth temperate forest of Patagonia, Argentina. *Ecosystems* **16**, 1025–1038 (2013).

25. Kandeler, E., Kampichler, C., Joergensen, R. G. & Mölter, K. Effects of mesofauna in a spruce forest on soil microbial communities and N cycling in field mesocosms. *Soil Biol. Biochem.* **31**, 1783–1792 (1999).

26. Salmon, S., Frizzera, L. & Camaret, S. Linking forest dynamics to richness and assemblage of soil zoological groups and to soil mineralization processes. *For. Ecol. Manag.* **256**, 1612–1623 (2009).

27. Wang, S., Ruan, H. & Wang, B. Effects of soil microarthropods on plant litter decomposition across an elevation gradient in the Wuyi Mountains. *Soil Biol. Biochem.* **41**, 891–897 (2009).

28. Frouz, J. Use of soil dwelling Diptera (Insecta, Diptera) as bioindicators: A review of ecological requirements and response to disturbance. *Agri. Ecos. Environ.* **74**, 167–186 (1999).

29. Anderson, J. M., Ineson, P. & Huish, S. A. Nitrogen and cation mobilization by soil fauna feeding on leaf litter and soil organic matter from deciduous woodlands. *Soil Biol. Biochem.* **15**, 463–467 (1983).

30. Eggleton, P. Global Patterns of Termite Diversity in *Termites: Evolution, Sociality, Symbioses, Ecology* (eds. Abe, T., Bignell, D. E. and Higashi, M.) 25–51 (Springer Netherlands, 2000). doi:10.1007/978-94-017-3223-9_2.

31. Sanderson, M. S. Biomass of termites and their emissions of methane and carbon dioxide: A global database. *Global Biogeochem. Cycles* **10**, 543–557 (1996).

32. Folgarait, P. J. *et al.* Soil properties and the macrofauna community in abandoned irrigated rice fields of northeastern Argentina. *Biol. Fert. Soil* **38**, 349–357 (2003).

33. Andrés, P. *et al.* Soil food web stability in response to grazing in a semi-arid prairie: The importance of soil textural heterogeneity. *Soil Biol. Biochem.* **97**, 131–143 (2016).

34. Bardgett, R. D. & Cook, R. Functional aspects of soil animal diversity in agricultural grasslands. *Appl. Soil Ecol.* **10**, 263–276 (1998).

35. Hendrix, P. F. *et al.* Detritus Food Webs in Conventional and No-Tillage Agroecosystems. *BioScience* **36**, 374–380 (1986).

36. Hunt, H. W. *et al.* The detrital foodweb in a shortgrass prairie. *Biol. Fert. Soils* **3**, 57–68 (1987).

37. Andriuzzi, W. S., Pulleman, M. M., Cluzeau, D. & Pérès, G. Comparison of two widely used sampling methods in assessing earthworm community responses to agricultural intensification. *Appl. Soil Ecol.* **119**, 145–151 (2017).

38. Birkhofer, K. *et al.* Soil fauna feeding activity in temperate grassland soils increases with legume and grass species richness. *Soil Biol. Biochem.* **43**, 2200–2207 (2011).

39. Briones, M. J. I., Ostle, N. J., McNamara, N. P. & Poskitt, J. Functional shifts of grassland soil communities in response to soil warming. *Soil Biol. Biochem.* **41**, 315–322 (2009).

40. Davidson, D. A. & Grieve, I. C. Relationships between biodiversity and soil structure and function: Evidence from laboratory and field experiments. *Appl. Soil Ecol.* **33**, 176–185 (2006).

41. Heděnec, P. *et al.* The effect of native and introduced biofuel crops on the composition of soil biota communities. *Biomass Bioenerg.* **60**, 137–146 (2014).

42. Heděnec, P. *et al.* Effect of long term cropping hybrid sorrel (Rumex patientia x Rumex tianshanicus) on soil biota. *Biomass Bioenerg.* **78**, 92–98 (2015).

43. Hirsch, P. R. *et al.* Starving the soil of plant inputs for 50 years reduces abundance but not diversity of soil bacterial communities. *Soil. Biol. Biochem.* **41**, 2021–2024 (2009).

44. Hirsch, P. R. *et al.* Soil resilience and recovery: rapid community responses to management changes. *Plant Soil* **412**, 283–297 (2017).

45. Niklaus, P. a. *et al.* Six years of in situ CO_2_ enrichment evoke changes in soil structure and soil biota of nutrient-poor grassland. *Glob. Change Biol.* 585–600 (2003) doi:10.1046/j.1365-2486.2003.00614.x.

46. Begum, F., Bajracharya, R. M., Sitaula, B. K. & Sharma, S. Seasonal dynamics, slope aspect and land use effects on soil mesofauna density in the mid-hills of Nepal. *Int. J. Biodiv. Sci. Ecos. Serv. Manag.* **9**, 290–297 (2013).

47. Brussaard  Bouwman L.A., Geurs M., Hassink J. & Zwart K.B., L. Biomass, composition and temporal dynamics of soil organisms of a silt loam soil under conventional and integrated management. *Netherl. Jour. Agricult. Sci.* **38**, 283–302 (1990).

48. Höfer, H. *et al.* Structure and function of soil fauna communities in Amazonian anthropogenic and natural ecosystems. *Eur. J. Soil Biol.* **37**, 229–235 (2001).

49. Araujo, P. I., Yahdjian, L. & Austin, A. T. Do soil organisms affect aboveground litter decomposition in the semiarid Patagonian steppe, Argentina? *Oecologia* **168**, 221–230 (2012).

50. Doblas-Miranda, E., Sánchez-Piñero, F. & González-Megías, A. Soil macroinvertebrate fauna of a Mediterranean arid system: Composition and temporal changes in the assemblage. *Soil Biol. Biochem.* **39**, 1916–1925 (2007).

51. El-Duweini, A. K. & Ghabbour, S. I. Population Density and Biomass of Earthworms in Different Types of Egyptian Soils. *Journal of Applied Ecology* **2**, 271–287 (1965).

52. Andrés, P., Mateos, E., Tarrasón, D., Cabrera, C. & Figuerola, B. Effects of digested, composted, and thermally dried sewage sludge on soil microbiota and mesofauna. *Appl. Soil. Ecol.*  **48**, 236–242 (2011).

53. Abensperg-Traun, M. *Termites (Isoptera) in Western Australia: present and future directions of ecological research*. (1998).

54. Ghabbour, S. I. & Mikhail, W. Z. A. Soil mesofauna in unstable environments and their population density-biomass relationship: Cases from the Egyptian deserts. *Geobios* **30**, 135–144 (1997).

55. Ueckert, D. N., Bodine, M. C. & Spears, B. M. Population density and biomass of the desert termite Gnathamitermes Tubiformans (Isoptera: Termitidae) in a Shortgrass Prairie: Relationship to Temperature and Moisture. vol. 57 (1976).

56. Blanchart, E. *et al.* Effect of direct seeding mulch-based systems on soil carbon storage and macrofauna in Central Brazil. *Agri. Con. Sci.* **72**, 81–87 (2007).

57. Djigal, D., Saj, S., Rabary, B., Blanchart, E. & Villenave, C. Mulch type affects soil biological functioning and crop yield of conservation agriculture systems in a long-term experiment in Madagascar. *Soil Till. Res.* **118**, 11–21 (2012).

58. Ayuke, F. O. *et al.* Soil fertility management: Impacts on soil macrofauna, soil aggregation and soil organic matter allocation. *Appl. Soil Ecol.* **48**, 53–62 (2011).

59. Jiménez, J. J. *et al.* Earthworm communities in native savannas and man-made pastures of the Eastern Plains of Colombia. *Biol. Fert. Soil* **28**, 101–110 (1998).

60. Jiménez, J. J., Decaëns, T. & Rossi, J. P. Stability of the spatio-temporal distribution and niche overlap in neotropical earthworm assemblages. *Acta Oecologica* **30**, 299–311 (2006).

61. Wong, M. K. *et al.* Comparison of soil macro-invertebrate communities in Malaysian oil palm plantations with secondary forest from the viewpoint of litter decomposition. *For. Ecol. Manag.* **381**, 63–73 (2016).

62. Franco, A. L. C. *et al.* Loss of soil (macro)fauna due to the expansion of Brazilian sugarcane acreage. *Sci. Tot. Env.* **563–564**, 160–168 (2016).

63. Marichal, R. *et al.* Soil macroinvertebrate communities and ecosystem services in deforested landscapes of Amazonia. *Appl. Soil Ecol.* **83**, 177–185 (2014).

64. Rousseau, G. X., Deheuvels, O., Rodriguez Arias, I. & Somarriba, E. Indicating soil quality in cacao-based agroforestry systems and old-growth forests: The potential of soil macrofauna assemblage. *Ecol. Indic.* **23**, 535–543 (2012).

65. Tsukamoto, J. & Sabang, J. Soil macro-fauna in an Acacia mangium plantation in comparison to that in a primary mixed dipterocarp forest in the lowlands of Sarawak, Malaysia. *Pedobiologia* **49**, 69–80 (2005).

66. Velasquez, E., Lavelle, P. & Andrade, M. GISQ, a multifunctional indicator of soil quality. *Soil. Biol. Biochem.* **39**, 3066–3080 (2007).

67. Yang, X. & Chen, J. Plant litter quality influences the contribution of soil fauna to litter decomposition in humid tropical forests, southwestern China. *Soil. Biol. Biochem.* **41**, 910–918 (2009).

68. Yang, X., Warren, M. & Zou, X. Fertilization responses of soil litter fauna and litter quantity, quality, and turnover in low and high elevation forests of Puerto Rico. *Aool. Soil. Ecol.* **37**, 63–71 (2007).

69. Vasconcellos, R. L. F., Segat, J. C., Bonfim, J. A., Baretta, D. & Cardoso, E. J. B. N. Soil macrofauna as an indicator of soil quality in an undisturbed riparian forest and recovering sites of different ages. *Eur. J. Soil. Biol.* **58**, 105–112 (2013).

70. Fittkau, E. J. & Klinge, H. On biomass and trophic structure of the Central Amazonian Rain Forest Ecosystem. *Biotropica* **5**, 2–14 (1973).

Table S2: Annual litterfall (kg ha^-1^ yr^-1^), total litterstock (kg ha^-1^), turnover ratio and turnover time across global biomes

| Biom | Annual litterfall (Mean ± SE) | n | Reference |
| --- | --- | --- | --- |
| Tundra and cold steppe | 1,702±706 | 9 | ^1–4^ |
| Boreal forest | 2,032±1,094 | 118 | ^2,5–7^ |
| Temperate forest | 3,221±1,394 | 283 | ^1,2,8–10^ |
| Temperate grassland | 2,997±1,053 | 19 | ^1–3,10,11^ |
| Mediterranean vegetation | 2,974±1,480 | 38 | ^1,2,12–17^ |
| Desert and semidesert | 638±448 | 12 | ^1,2,12,13,16,18,19^ |
| Tropical grassland | 3,893±1,894 | 36 | ^1,2,16,20–22^ |
| Tropical forest | 5,413±1,394 | 182 | ^1,2,9,16,23,24^ |
|  |  |  |  |
| Biom | Annual litter stock (Mean ± SE) | n | Reference |
| Tundra and cold steppe | 5,210±2,946 | 7 | ^1–4^ |
| Boreal forest | 8,925±4,758 | 10 | ^2,5–7^ |
| Temperate forest | 11,112±6,345 | 88 | ^1,2,8–10^ |
| Temperate grassland | 2,500±707 | 6 | ^1–3,10,11^ |
| Mediterranean vegetation | 5,318±3090 | 14 | ^1,2,12–17^ |
| Desert and semidesert | 918±698 | 5 | ^1,2,12,13,16,18,19^ |
| Tropical grassland | 3,378±1990 | 17 | ^1,2,16,20–22^ |
| Tropical forest | 6,300±3366 | 46 | ^1,2,9,16,23,24^ |
|  |  |  |  |
| Biom | Fine root biomass (Mean ± SE) | n | Reference |
| Tundra and cold steppe | 9,600±2,200 | 5 | ^25,26^ |
| Boreal forest | 6,000±1,300 | 5 | ^25,26^ |
| Temperate forest | 8,000±1,500 | 24 | ^25,26^ |
| Temperate grassland | 15,700±1,400 | 21 | ^25,26^ |
| Mediterranean vegetation | 5,200±1,300 | 6 | ^25,26^ |
| Desert and semidesert | 2,700±1,000 | 5 | ^25,26^ |
| Tropical grassland | 9,900±2,400 | 5 | ^25,26^ |
| Tropical forest | 5,700±1,500 | 18 | ^25,26^ |
|  |  |  |  |
| Biom | Turnover rate±SE (litterfall/litterstock) | Turnover time±SE(litterstock/litterfall) | |
| Tundra and cold steppe | 0.327±0.24 | 3.061±4.175 |  |
| Boreal forest | 0.228±0.23 | 4.393±4.349 |  |
| Temperate forest | 0.29±0.22 | 3.45±4.550 |  |
| Temperate grassland | 1.199±1.489 | 0.834±0.671 |  |
| Mediterranean vegetation | 0.559±0.479 | 1.788±2.088 |  |
| Desert and semidesert | 0.695±0.642 | 1.438±1.55 |  |
| Tropical grassland | 1.152±0.952 | 0.868±1.051 | |
| Tropical forest | 0.859±0.414 | 1.164±2.414 | |

The n reffers number of observations

Database Holland et al.^2^ includes data from 685 original literature sources dating from 1827 to 1997.

Database Jackson et al.^25,26^ includes data from 250 original literature sources dating from 1950 to 1995.

**References:**

1. Brovkin, V. *et al.* Plant-driven variation in decomposition rates improves projections of global litter stock distribution. *Biogeosciences* **9**, 565–576 (2012).

2. Holland, E. A. *et al.* A global database of litterfall mass and litter pool carbon and nutrients. (2014) doi:10.3334/ORNLDAAC/1244.

3. Palpurina, S. *et al.* The type of nutrient limitation affects the plant species richness–productivity relationship: Evidence from dry grasslands across Eurasia. *J. Ecol.* **107**, 1038–1050 (2019).

4. Shchelchkova, M., Davydov, S., Fyodorov-Davydov, D., Davydova, A. & Boeskorov, G. The characteristics of a relic steppe of northeast asia: refuges of the pleistocene mammoth steppe (an example from the Lower Kolyma area). *IOP Conference Series: Earth and Environmental Science* **438**, (2020).

5. Kurka, A. M., Starr, M., Heikinheimo, M. & Salkinoja-Salonen, M. Decomposition of cellulose strips in relation to climate, litterfall nitrogen, phosphorus and C/N ratio in natural boreal forests. *Plant Soil* **219**, 91–101 (2000).

6. Matala, J., Kellomaki, S. & Nuutinen, T. Litterfall in relation to volume growth of trees: Analysis based on literature. *Scand. J. For. Res.* **23**, 194–202 (2008).

7. Miller, H. G. Dynamics of nutrient cycling in plantation ecosystems in *Nutrition of plantation forests* 54-78 (1984).

8. Clark, D. A. *et al.* Net primary production in tropical forests : An evaluation and synthesis of existing field **11**, 371–384 (2001).

9. Yanai, R. D. *et al.* Litterfall and litter chemistry change over time in an old-growth temperate forest, northeastern China. *For. Ecol. Manag.* **43**, 279–287 (1999).

10. Pedersen, L. B. & Bille-Hansen, J. A comparison of litterfall and element fluxes in even aged Norway spruce, sitka spruce and beech stands in Denmark. *For. Ecol. Manag.* **114**, 55–70 (1999).

11. Li, X., Liu, B., Chen, L. & Song, N. Effects of litter accumulation on plant communities in fenced desert steppe. *Pol. J. of Ecol.* **63**, 333–340 (2015).

12. Alvarez, J. A., Villagra, P. E., Rossi, B. E. & Cesca, E. M. Spatial and temporal litterfall heterogeneity generated by woody species in the Central Monte desert. *Plant Ecol.* **205**, 295–303 (2009).

13. Búrquez, A., Martínez-Yrízar, A. & Núñez, S. Sonoran Desert productivity and the effect of trap size on litterfall estimates in dryland vegetation. *J. Arid Env.* **43**, 459–465 (1999).

14. Cappai, C. *et al.* Small-scale spatial variation of soil organic matter pools generated by cork oak trees in Mediterranean agro-silvo-pastoral systems. *Geoderma* **304**, 59–67 (2017).

15. La Mantia, T. *et al.* The effects of post-pasture woody plant colonization on soil and aboveground litter carbon and nitrogen along a bioclimatic transect. *IForest* **6**, 238–246 (2013).

16. Nelson, M. Litterfall and decomposition rates in Biosphere 2 terrestrial biomes. *Ecol. Eng.* **13**, 135–145 (1999).

17. Sferlazza, S. *et al.* Effects of traditional forest management on carbon storage in a Mediterranean holm oak (Quercus ilex L.) coppice. *IForest* **11**, 344–351 (2018).

18. Kulakova, N. Impact of plant species on the formation of carbon and nitrogen stock in soils under semi-desert conditions. *Eur. J. For. Res.* **131**, 1717–1726 (2012).

19. Martínez-Yrízar, A., Núñez, S. & Búrquez, A. Leaf litter decomposition in a southern Sonoran Desert ecosystem, northwestern Mexico: Effects of habitat and litter quality. *A. Oecologica* **32**, 291–300 (2007).

20. Bennett, L. T., Judd, T. S. & Adams, M. A. Growth and nutrient content of perennial grasslands following burning in semi-arid, sub-tropical Australia. *Plant Ecol.* **164**, 185–199 (2003).

21. González-Roglich, M., Swenson, J. J., Jobbágy, E. G. & Jackson, R. B. Shifting carbon pools along a plant cover gradient in woody encroached savannas of central Argentina. *For. Ecol. Manag.* **331**, 71–78 (2014).

22. Mlambo, D. & Nyathi, P. Litterfall and nutrient return in a semi-arid southern African savanna woodland dominated by Colophospermum mopane. *Plant Ecol.* **196**, 101–110 (2008).

23. Parsons, S. A., Congdon, R. A., Shoo, L. P., Valdez-Ramirez, V. & Williams, S. E. Spatial variability in litterfall, litter standing crop and litter quality in a tropical rain forest region. *Biotropica* **46**, 378–386 (2014).

24. Santos, F. M., Terra, G., Piotto, D. & Chaer, G. M. Recovering ecosystem functions through the management of regenerating community in agroforestry and plantations with Khaya spp. in the Atlantic Forest, Brazil. *For. Ecol. Manag.* **482**, 118854 (2021).

25. Jackson, R. B. *et al.* A global analysis of root distributions for terrestrial biomes. *Oecologia* **108**, 389–411 (1996).

26. Jackson, R. B., Mooney, H. A. & Schulze, E.-D. A global budget for fine root biomass, surface area, and nutrient contents. *PNAS* **94**, 7362–7366 (1997).

Table S3: Average litter consumption by soil fauna expressed as consumed dry weight of litter per dry biomass of animal per day

| Species | Taxonomic groups | Guild | Assumption | Mean consumption (mg dw. mg d bodymass^-1^.d^-1^)±SD | | Reference |
| --- | --- | --- | --- | --- | --- | --- |
| *Aamara aenea* | Carabidae | Herbivores | 20% dry matter content for fauna^1^ | 2.115 | | ^1^ |
| *Amara aenea* | Carabidae | Herbivores | 20% dry matter content for fauna | | 3.310 | ^1^ |
| *Amara aulica* | Carabidae | Herbivores | 20% dry matter content for fauna | | 1.575 | ^1^ |
| *Amara aulica* | Carabidae | Herbivores | 20% dry matter content for fauna | | 0.035 | ^1^ |
| *Amara consulatis* | Carabidae | Herbivores | 20% dry matter content for fauna | | 0.335 | ^1^ |
| *Amara consulatis* | Carabidae | Herbivores | 20% dry matter content for fauna | | 0.015 | ^1^ |
| *Amara familiaris* | Carabidae | Herbivores | 20% dry matter content for fauna | | 0.275 | ^1^ |
| *Amara familiaris* | Carabidae | Herbivores | 20% dry matter content for fauna | | 2.300 | ^1^ |
| *Amara littoreta* | Carabidae | Herbivores | 20% dry matter content for fauna | | 2.245 | ^1^ |
| *Amara littoreta* | Carabidae | Herbivores | 20% dry matter content for fauna | | 4.045 | ^1^ |
| *Amara ovata* | Carabidae | Herbivores | 20% dry matter content for fauna | | 1.495 | ^1^ |
| *Amara ovata* | Carabidae | Herbivores | 20% dry matter content for fauna | | 1.440 | ^1^ |
| *Amara similata* | Carabidae | Herbivores | 20% dry matter content for fauna | | 1.850 | ^1^ |
| *Amara similata* | Carabidae | Herbivores | 20% dry matter content for fauna | | 3.625 | ^1^ |
| *Anchomenus dorsalis* | Carabidae | Herbivores | 20% dry matter content for fauna | | 0.470 | ^1^ |
| *Anisodactylus signatus* | Carabidae | Herbivores | 20% dry matter content for fauna | | 2.295 | ^1^ |
| *Calathus ambiguus* | Carabidae | Herbivores | 20% dry matter content for fauna | | 0.500 | ^1^ |
| *Calathus ambiguus* | Carabidae | Herbivores | 20% dry matter content for fauna | | 1.035 | ^1^ |
| *Calathus fucipes* | Carabidae | Herbivores | 20% dry matter content for fauna | | 0.025 | ^1^ |
| *Dolichus halensis* | Carabidae | Herbivores | 20% dry matter content for fauna | | 0.030 | ^1^ |
| *Dolichus halensis* | Carabidae | Herbivores | 20% dry matter content for fauna | | 0.020 | ^1^ |
| *Harpalus affinis* | Carabidae | Herbivores | 20% dry matter content for fauna | | 2.640 | ^1^ |
| *Harpalus affinis* | Carabidae | Herbivores | 20% dry matter content for fauna | | 1.135 | ^1^ |
| *Harpalus atratus* | Carabidae | Herbivores | 20% dry matter content for fauna | | 0.690 | ^1^ |
| *Harpalus signaticornis* | Carabidae | Herbivores | 20% dry matter content for fauna | | 2.225 | ^1^ |
| *Harpalus signaticornis* | Carabidae | Herbivores | 20% dry matter content for fauna | | 2.135 | ^1^ |
| *Harpalus tardus* | Carabidae | Herbivores | 20% dry matter content for fauna | | 1.940 | ^1^ |
| *Harpalus tardus* | Carabidae | Herbivores | 20% dry matter content for fauna | | 0.350 | ^1^ |
| *Harpalys distinguendus* | Carabidae | Herbivores | 20% dry matter content for fauna | | 1.610 | ^1^ |
| *Harpalys distinguendus* | Carabidae | Herbivores | 20% dry matter content for fauna | | 1.350 | ^1^ |
| *Heteronychus arator* | Carabidae | Herbivores | NA | | 0.093 | ^2^ |
| *Heteronychus arator* | Carabidae | Herbivores | NA | | 0.026 | ^2^ |
| *Ophonus azureus* | Carabidae | Herbivores | 20% dry matter content for fauna | | 0.640 | ^1^ |
| *Ophonus azureus* | Carabidae | Herbivores | 20% dry matter content for fauna | | 0.540 | ^1^ |
| *Poecilus cuperus* | Carabidae | Herbivores | 20% dry matter content for fauna | | 0.425 | ^1^ |
| *Poecilus cuperus* | Carabidae | Herbivores | 20% dry matter content for fauna | | 0.025 | ^1^ |
| *Pseudoophonus rufipes* | Carabidae | Herbivores | 20% dry matter content for fauna | | 2.095 | ^1^ |
| *Pseudoophonus rufipes* | Carabidae | Herbivores | 20% dry matter content for fauna | | 0.065 | ^1^ |
| *Pterostichus melanarius* | Carabidae | Herbivores | 20% dry matter content for fauna | | 0.155 | ^1^ |
| *Trechus quadristiatus* | Carabidae | Herbivores | 20% dry matter content for fauna | | 1.505 | ^1^ |
| Mean Herbivores | |  |  | | 1.217±1.1 |  |
| *Cylindroiulus caeruleocinctus* | Diplopoda | Litter feeders | NA | | 0.037 | ^3^ |
| *Allaiulus fuhiceps* | Diplopoda | Litter feeders | 20% dry matter content for fauna + 25% dry matter content for litter | | 0.363 | ^4^ |
| *Allaiulus fulviceps* | Diplopoda | Litter feeders | NA | | 0.363 | ^4^ |
| *Allajulus latestriatus* | Diplopoda | Litter feeders | 20% dry matter content for fauna | | 0.025 | ^5^ |
| *Allajulus latestriatus* | Diplopoda | Litter feeders | 20% dry matter content for fauna | | 0.020 | ^5^ |
| *Allajulus latestriatus* | Diplopoda | Litter feeders | 20% dry matter content for fauna | | 0.017 | ^5^ |
| *Anadenus altivagus* | Mollusca | Litter feeders | NA | | 0.011 | ^6^ |
| *Arianta arbustorum* | Mollusca | Litter feeders | NA | | 0.018 | ^7^ |
| *Armadillidium vulgare* | Isopoda | Litter feeders | NA | | 0.175 | ^8^ |
| *Balloniscus sellowii* | Isopoda | Litter feeders | 20% dry matter content for fauna | | 0.265 | ^9^ |
| *Balloniscus sellowii* | Isopoda | Litter feeders | 20% dry matter content for fauna | | 0.075 | ^9^ |
| *Balloniscus sellowii* | Isopoda | Litter feeders | 20% dry matter content for fauna | | 0.155 | ^9^ |
| *Balloniscus sellowii* | Isopoda | Litter feeders | 20% dry matter content for fauna | | 0.205 | ^9^ |
| *Balloniscus sellowii* | Isopoda | Litter feeders | 20% dry matter content for fauna | | 0.260 | ^9^ |
| *Balloniscus sellowii* | Isopoda | Litter feeders | 20% dry matter content for fauna | | 0.400 | ^9^ |
| *Balloniscus sellowii* | Isopoda | Litter feeders | 20% dry matter content for fauna | | 0.267 | ^9^ |
| *Balloniscus sellowii* | Isopoda | Litter feeders | NA | | 0.167 | ^9^ |
| *Bibio marci* | Diptera | Litter feeders | NA | | 0.150 | ^10^ |
| *Bibio pomonae* | Diptera | Litter feeders | NA | | 0.205 | ^11^ |
| *Binio pomonae* | Diptera | Litter feeders | NA | | 0.320 | ^11^ |
| *Cylindroiulus bursenlandicus* | Diplopoda | Litter feeders | 20% dry matter content for fauna | | 0.407 | ^12^ |
| *Cylindrojulus boleti* | Diplopoda | Litter feeders | NA | | 0.035 | ^13^ |
| *Cylindrojulus luridus* | Diplopoda | Litter feeders | NA | | 0.035 | ^13^ |
| *Discus rotundatus* | Molluscsa | Litter feeders | NA | | 0.007 | ^7^ |
| *Eudrious pulchripes,* | Diplopoda | Litter feeders | NA | | 0.012 | ^14^ |
| *Eudrilus eugeniae* | Earthworm | Litter feeders | NA | | 0.064 | ^15^ |
| *Glomeris hexastica* | Diplopoda | Litter feeders | NA | | 0.037 | ^3^ |
| *Glomeris hexasticha* | Diplopoda | Litter feeders | 20% dry matter content for fauna | | 0.982 | ^16^ |
| *Glomeris hexasticha* | Diplopoda | Litter feeders | 20% dry matter content for fauna | | 0.901 | ^16^ |
| *Glomeris hexasticha* | Diplopoda | Litter feeders | 20% dry matter content for fauna | | 0.545 | ^16^ |
| *Glomeris hexasticha* | Diplopoda | Litter feeders | NA | | 0.076 | ^13^ |
| *Glomeris marginata* | Diplopoda | Litter feeders | 20% dry matter content for fauna | | 0.192 | ^17^ |
| *Glomeris marginata* | Diplopoda e | Litter feeders | 20% dry matter content for fauna | | 0.142 | ^17^ |
| *Glomeris marginata* | Diplopoda | Litter feeders | NA | | 0.423 | ^18^ |
| *Glomeris marginata* | Diplopoda | Litter feeders | NA | | 0.384 | ^18^ |
| *Glomeris marginata* | Diplopoda | Litter feeders | NA | | 0.172 | ^18^ |
| *Glomeris marginata* | Diplopoda | Litter feeders | NA | | 0.119 | ^18^ |
| *Glomeris marginata* | Diplopoda | Litter feeders | NA | | 0.111 | ^19^ |
| *Harpaphe haydeniana* | Diplopoda | Litter feeders | 20% dry matter content for fauna + 25% dry matter content for litter | | 0.388 | ^20^ |
| *Harpaphe haydeniana* | Diplopoda | Litter feeders | 20% dry matter content for fauna + 25% dry matter content for litter | | 0.325 | ^20^ |
| *Harpaphe haydeniana* | Diplopoda | Litter feeders | 20% dry matter content for fauna + 25% dry matter content for litter | | 0.213 | ^20^ |
| Harpaphe haydeniana | Diplopoda | Litter feeders | 20% dry matter content for fauna + 25% dry matter content for litter | | 0.263 | ^20^ |
| *Harpaphe haydeniana* Juvenile | Diplopoda | Litter feeders | 20% dry matter content for fauna + 25% dry matter content for litter | | 0.338 | ^20^ |
| *Harpaphe haydeniana* Juvenile | Diplopoda | Litter feeders | 20% dry matter content for fauna + 25% dry matter content for litter | | 0.338 | ^20^ |
| *Harpaphe haydeniana* Juvenile | Diplopoda | Litter feeders | 20% dry matter content for fauna + 25% dry matter content for litter | | 0.363 | ^20^ |
| *Harpaphe haydeniana* Juvenile | Diplopoda | Litter feeders | 20% dry matter content for fauna + 25% dry matter content for litter | | 0.350 | ^20^ |
| *Chromatoiulus projectus* | Diplopoda | Litter feeders | 20% dry matter content for fauna | | 0.320 | ^20^ |
| *Chromatojulus projectus* | Diplopoda | Litter feeders | NA | | 0.056 | ^13^ |
| *Chromatojulus unilineatus* | Diplopoda | Litter feeders | NA | | 0.024 | ^13^ |
| *Leptoiulus proximus* | Diplopoda | Litter feeders | NA | | 0.020 | ^13^ |
| *Leptoiulus trilobatus* | Diplopoda | Litter feeders | 20% dry matter content for fauna | | 0.752 | ^12^ |
| *Narceus annularis* | Diplopoda | Litter feeders | NA | | 0.362 | ^21^ |
| *Ommatoiulus sabulosus* | Diplopoda | Litter feeders | 20% dry matter content for fauna | | 0.529 | ^4^ |
| *Ommatoiulus sabulosus* | Diplopoda | Litter feeders | NA | | 0.529 | ^4^ |
| *Oniscus asellus* | Isopoda | Litter feeders | 20% dry matter content for fauna | | 0.160 | ^22^ |
| *Oniscus asellus* | Isopoda | Litter feeders | 20% dry matter content for fauna | | 0.015 | ^22^ |
| *Oniscus asellus* | Isopoda | Litter feeders | 20% dry matter content for fauna | | 0.070 | ^22^ |
| *Oniscus asellus* | Isopoda | Litter feeders | 20% dry matter content for fauna | | 0.030 | ^22^ |
| *Oniscus asellus* | Isopoda | Litter feeders | 20% dry matter content for fauna | | 0.030 | ^22^ |
| *Oniscus asellus* | Isopoda | Litter feeders | 20% dry matter content for fauna | | 0.020 | ^22^ |
| *Oniscus asellus* | Isopoda | Litter feeders | 20% dry matter content for fauna | | 0.040 | ^23^ |
| *Oniscus asellus* | Isopoda | Litter feeders | 20% dry matter content for fauna | | 0.060 | ^23^ |
| *Oniscus asellus* | Isopoda | Litter feeders | 20% dry matter content for fauna | | 0.045 | ^23^ |
| *Oniscus asellus* | Isopoda | Litter feeders | 20% dry matter content for fauna | | 0.055 | ^23^ |
| *Ophiiulus pilosus* | Diplopoda | Litter feeders | NA | | 0.516 | ^4^ |
| *Ophyiulus pilosus* | Diplopoda | Litter feeders | 20% dry matter content for fauna | | 0.516 | ^4^ |
| *Orthomorpha gracilis* | Diplopoda | Litter feeders | NA | | 6.040 | ^24^ |
| *Oxychilus cellarius* | Molluscsa | Litter feeders | NA | | 0.008 | ^7^ |
| *Parafontaria pruinosus* | Isopoda | Litter feeders | 20% dry matter content for fauna | | 0.027 | ^25^ |
| *Parafontaria pruinosus* | Isopoda | Litter feeders | 20% dry matter content for fauna | | 0.063 | ^25^ |
| *Parafontaria laminata* | Diplopoda | Litter feeders | NA | | 0.625 | ^26^ |
| *Penthetria holosericea* | Diptera | Litter feeders | NA | | 0.650 | ^10^ |
| *Penthetria holosericea* | Diptera | Litter feeders | NA | | 0.070 | ^10^ |
| *Porcelio dilatatus* | Isopoda | Litter feeders | NA | | 0.185 | ^27^ |
| *Porcelio scaber* | Isopoda | Litter feeders | NA | | 0.134 | ^3^ |
| *Porcellio scaber* | Isopoda | Litter feeders | NA | | 0.009 | ^28^ |
| *Porcellio scaber* | Isopoda | Litter feeders | 20% dry matter content for fauna | | 0.024 | ^25^ |
| *Porcellio scaber* | Isopoda | Litter feeders | 20% dry matter content for fauna | | 0.038 | ^25^ |
| *Porcellio scaber* | Isopoda | Litter feeders | 20% dry matter content for fauna | | 0.370 | ^22^ |
| *Porcellio scaber* | Isopoda | Litter feeders | 20% dry matter content for fauna | | 0.045 | ^22^ |
| *Porcellio scaber* | Isopoda | Litter feeders | 20% dry matter content for fauna | | 0.105 | ^22^ |
| *Porcellio scaber* | Isopoda | Litter feeders | 20% dry matter content for fauna | | 0.035 | ^22^ |
| *Porcellio scaber* | Isopoda | Litter feeders | 20% dry matter content for fauna | | 0.040 | ^22^ |
| *Porcellio scaber* | Isopoda | Litter feeders | 20% dry matter content for fauna | | 0.145 | ^22^ |
| *Porcellio scaber* | Isopoda | Litter feeders | 20% dry matter content for fauna | | 0.029 | ^29^ |
| *Porcellio scaber* | Isopoda | Litter feeders | 20% dry matter content for fauna | | 0.210 | ^30^ |
| *Porcellio scaber* | Isopoda | Litter feeders | 20% dry matter content for fauna | | 0.600 | ^30^ |
| *Porcellio scaber* | Isopoda | Litter feeders | 20% dry matter content for fauna | | 0.357 | ^30^ |
| *Porcellio scaber* | Isopoda | Litter feeders | 20% dry matter content for fauna | | 0.211 | ^30^ |
| *Porcellio scaber* | Isopoda | Litter feeders | 20% dry matter content for fauna | | 0.052 | ^31^. |
| *Porcellio scaber* | Isopoda | Litter feeders | 20% dry matter content for fauna | | 0.015 | ^31^. |
| *Porcellionides pruinosus* | Isopoda | Litter feeders | 20% dry matter content for fauna | | 0.377 | ^32^ |
| *Porcellionides pruinosus* | Isopoda | Litter feeders | 20% dry matter content for fauna | | 0.348 | ^32^ |
| *Porcellionides pruinosus* | Isopoda | Litter feeders | 20% dry matter content for fauna | | 0.198 | ^32^ |
| *Porcellionides pruinosus* | Isopoda | Litter feeders | 20% dry matter content for fauna | | 0.200 | ^32^ |
| *Porcellionides pruinosus* | Isopoda | Litter feeders | 20% dry matter content for fauna | | 0.600 | ^30^ |
| *Porcellionides pruinosus* | Isopoda | Litter feeders | 20% dry matter content for fauna | | 0.357 | ^30^ |
| *Porcellionides pruinosus* | Isopoda | Litter feeders | 20% dry matter content for fauna | | 0.357 | ^30^ |
| *Porcellionides pruinosus* | Isopoda | Litter feeders | 20% dry matter content for fauna | | 0.357 | ^30^ |
| *Rossiulus kessleri* | Diplopoda | Litter feeders | 20% dry matter content for fauna | | 3.750 | ^33^ |
| *Rossiulus kessleri* | Diplopoda | Litter feeders | 20% dry matter content for fauna | | 2.500 | ^33^ |
| *Rossiulus kessleri* | Diplopoda | Litter feeders | 20% dry matter content for fauna | | 1.800 | ^33^ |
| *Rossiulus kessleri* | Diplopoda | Litter feeders | 20% dry matter content for fauna | | 1.750 | ^33^ |
| *Strongilosoma pallipes* | Diplopoda | Litter feeders | NA | | 0.035 | ^13^ |
| *Traceolipus aoutii* | Diplopoda | Litter feeders | NA | | 0.080 | ^14^ |
| *Traceolipus radkei* | Isopoda | Litter feeders | NA | | 0.550 | ^34^ |
| *Traceolipus radkei* | Isopoda | Litter feeders | NA | | 0.689 | ^34^ |
| *Traceolipus radkei* | Isopoda | Litter feeders | NA | | 0.517 | ^34^ |
| *Traceolipus radkei* | Isopoda | Litter feeders | NA | | 0.454 | ^34^ |
| *Traceolipus radkei* | Isopoda | Litter feeders | NA | | 0.407 | ^34^ |
| *Trachelipus rathkii* | Isopoda | Litter feeders | 20% dry matter content for fauna | | 0.160 | ^22^ |
| *Trachelipus rathkii* | Isopoda | Litter feeders | 20% dry matter content for fauna | | 0.030 | ^22^ |
| *Trachelipus rathkii* | Isopoda | Litter feeders | 20% dry matter content for fauna | | 0.050 | ^22^ |
| *Trachelipus rathkii* | Isopoda | Litter feeders | 20% dry matter content for fauna | | 0.050 | ^22^ |
| *Trachelipus rathkii* | Isopoda | Litter feeders | 20% dry matter content for fauna | | 0.020 | ^22^ |
| *Trachelipus rathkii* | Isopoda | Litter feeders | 20% dry matter content for fauna | | 0.025 | ^22^ |
| *Trachelipus ratzeburgii* | Isopoda | Litter feeders | 20% dry matter content for fauna | | 0.405 | ^22^ |
| *Trachelipus ratzeburgii* | Isopoda | Litter feeders | 20% dry matter content for fauna | | 0.055 | ^22^ |
| *Trachelipus ratzeburgii* | Isopoda | Litter feeders | 20% dry matter content for fauna | | 0.030 | ^22^ |
| *Trachelipus ratzeburgii* | Isopoda | Litter feeders | 20% dry matter content for fauna | | 0.050 | ^22^ |
| *Trachelipus ratzeburgii* | Isopoda | Litter feeders | 20% dry matter content for fauna | | 0.070 | ^22^ |
| *Trachelipus ratzeburgii* | Isopoda | Litter feeders | 20% dry matter content for fauna | | 0.205 | ^22^ |
| *Trochulus hispidus* | Mollusca | Litter feeders | NA | | 0.016 | ^7^ |
| *Unciger foetidus* | Diplopoda | Litter feeders | 20% dry matter content for fauna | | 1.014 | ^12^ |
| *Unciger foetidus* | Diplopoda | Litter feeders | NA | | 0.013 | ^13^ |
| *Xyloryctes lobicollis* | Colleoptaera | Litter feeders | NA | | 0.026 | ^35^ |
| Litter feeders mean | |  |  | | 0.337±0.69 |  |
| *Allolobophora caliginosa* | Earthworm | Earthworm | 20% dry matter content for fauna | | 0.770 | ^36^ |
| *Allolobophora caliginosa* | Earthworm | Earthworm | 20% dry matter content for fauna | | 1.060 | ^36^ |
| *Allolobophora caliginosa* | Earthworm | Earthworm | 20% dry matter content for fauna | | 1.010 | ^36^ |
| *Allolobophora caliginosa* | Earthworm | Earthworm | 20% dry matter content for fauna | | 0.877 | ^36^ |
| *Allolobophora caliginosa* | Earthworm | Earthworm | 20% dry matter content for fauna | | 1.130 | ^36^ |
| *Allolobophora caliginosa* | Earthworm | Earthworm | 20% dry matter content for fauna | | 1.110 | ^36^ |
| *Allolobophora caliginosa* | Earthworm | Earthworm | 20% dry matter content for fauna | | 0.799 | ^36^ |
| *Allolobophora caliginosa* | Earthworm | Earthworm | 20% dry matter content for fauna | | 1.180 | ^36^ |
| *Allolobophora caliginosa* | Earthworm | Earthworm | 20% dry matter content for fauna | | 1.140 | ^36^ |
| combined both | Earthworm | Earthworm | 20% dry matter content for fauna | | 0.105 | ^37^ |
| combined both | Earthworm | Earthworm | 20% dry matter content for fauna | | 0.080 | ^37^ |
| combined both | Earthworm | Earthworm | 20% dry matter content for fauna | | 0.050 | ^37^ |
| *Dendrobaena platyura* | Earthworm | Earthworm | NA | | 0.073 | ^38^ |
| *Lumbricus rubellus* | Earthworm | Earthworm | NA | | 0.940 | ^38^ |
| *Lumbricus terrestris* | Earthworm | Earthworm | NA | | 0.163 | ^38^ |
| *Lumbricus. rubellus* | Earthworm | Earthworm | 20% dry matter content for fauna | | 0.065 | ^37^ |
| *Lumbricus rubellus* | Earthworm | Earthworm | 20% dry matter content for fauna | | 0.030 | ^37^ |
| *Lumbricus polyphemus* | Earthworm | Earthworm | NA | | 0.068 | ^38^ |
| *Lumbricus rubellus* | Earthworm | Earthworm | 20% dry matter content for fauna | | 0.115 | ^37^ |
| *Lumbricus terrestris* | Earthworm | Earthworm | NA | | 0.085 | ^39^ |
| *Lumbricus terrestris* | Earthworm | Earthworm | NA | | 0.083 | ^39^ |
| *Lumbricus terrestris* | Earthworm | Earthworm | NA | | 0.079 | ^39^ |
| *Lumbricus terrestris* | Earthworm | Earthworm | NA | | 0.065 | ^39^ |
| *Lumbricus terrestris* | Earthworm | Earthworm | NA | | 0.072 | ^38^ |
| *Octolasion lacteum* | Earthworm | Earthworm | 20% dry matter content for fauna | | 0.010 | ^37^ |
| *Octolasion lacteum* | Earthworm | Earthworm | 20% dry matter content for fauna | | 0.010 | ^37^ |
| *Octolasion lacteum* | Earthworm | Earthworm | 20% dry matter content for fauna | | 0.020 | ^37^ |
| Earthworms mean | |  |  | | 0.414±0.46 |  |
| *Coptotermes sormosanus* | Termite | Termites | termite weight Morales ramos pers com | | 0.174 | ^40^ |
| *Coptotermes sormosanus* | Termite | Termites | termite weight Morales ramos pers com | | 0.217 | ^40^ |
| *Reticulitermes flavipes* | Termite | Termites | termite weight | | 0.094 | ^41^ |
| *Termites community* | Termite | Termites | termite weight | | 0.046 | ^42^ |
| *Termites community* | Termite | Termites | termite weight | | 0.053 | ^43^ |
| *Trinervitermes trinervoides* | Termite | Termites | termite weight | | 0.017 | ^44^ |
| *Trinervitermes trinervoides* | Termite | Termites | termite weight | | 0.004 | ^44^ |
| Termites mean | |  |  | | 0.086±0.08 |  |

**References:**

1. Honěk, A., Martinkova, Z. & Jarosik, V. Ground beetles (Carabidae) as seed predators. *Eur. J. Ent.* **100**, 531–544 (2003).

2. King, P. D., Mercer, C. F. & Meekings, J. S. Ecology Of Black beetle *Heteronychus arator*, influence of plant species on larval consumption, utilization and growth. *Ent. Exp. Appl.* **29**, 109–116 (1981).

3. M. Ardestani, M., Šustr, V. & Frouz, J. Consumption Performance of Five Detritivore Species Feeding on Alnus glutinosa L. Leaf Litter in a Microcosm Experiment. *Forests* **10**, (2019).

4. Brüggl, G. Gut passage, respiratory rate and assimilation efficiency of three millipedes from a deciduous wood in the Alps. *Adv.Myr.* 319–325 (1992).

5. Weber, M. & Nentwig, W. Impact of Bt corn on the diplopod Allajulus latestriatus. *Pedobiologia* **50**, 357–368 (2006).

6. Gupta, P. K. & Oli, B. P. Consumption and assimilation of evergreen oak litter by the slug *Anadenus altivagus* in Kumaon Himalayan Forests, India. *Écoscience* **5**, 494–501 (1998).

7. Astor, T., Lenoir, L. & Berg, M. P. Measuring feeding traits of a range of litter-consuming terrestrial snails: leaf litter consumption, faeces production and scaling with body size. *Oecologia* **178**, 833–845 (2015).

8. David, J.-F., Malet, N., Coûteaux, M.-M. & Roy, J. Feeding rates of the woodlouse *Armadillidium vulgare* on herb litters produced at two levels of atmospheric CO_2_. *Oecologia* **127**, 343–349 (2001).

9. Wood, C. T., Schlindwein, C. C. D., Soares, G. L. G. & Araujo, P. B. Feeding rates of *Balloniscus sellowii* (Crustacea, Isopoda, Oniscidea): The effect of leaf litter decomposition and its relation to the phenolic and flavonoid content. *ZooKeys* **176**, 231–245 (2012).

10. Frouz, J. & Šustr, V. The impact of drying and rewetting of leaf litter on feeding activity of *Bibio pomonae* (Diptera, Bibionidae) larvae. *S. Dipter.* **3**, 101–106 (1996).

11. Frouz, J., Šustr, V. & Kalčík, J. Energetic budget of three species of bibionid larvae. *Contributions to Soil Zoology in Central Europe I. ISB AS CR, České Budějovice* 15–18 (2005).

12. Striganova, B. R. Dispersion patterns of diplopods and their activity in the litter decomposition in the Carpathian foothills. in *Progress in Soil Zoology: Proceedings of the 5th International Colloquium on Soil Zoology Held in Prague September 17--22, 1973* (ed. Vaněk, J.) 167–173 (Springer Netherlands, 1975). doi:10.1007/978-94-010-1933-0_19.

13. Pobozsny, M. Nahrungsanprueche einiger diplopoden und isopoden-arten in mesophilen laubwaeldern Ungarns. (1978).

14. Ardestani, M. M. Comparison Among Test Substrates in Metal Uptake and Toxicity to Folsomia candida and Hordeum vulgare. *B. Environ. Cont. Tox.* **104**, 400–410 (2020).

15. Kamdem, M. M., Ngakou, A., Yanou Njintang, N. & Voua Otomo, P. Habitat components and population density drive plant litter consumption by *Eudrilus eugeniae* (Oligochaeta) under tropical conditions. *Integr. Zool.***16**, 255–269 (2021).

16. Tajovsky, K. Feeding Biology of the Millipede *Glomeris hexasticha*. *Ber. nat.-med. Verein Innsbruck* **10**, 305–311 (1992).

17. David, J. F. & Gillon, D. Annual feeding rate of the millipede Glomeris marginata on holm oak (Quercus ilex) leaf litter under Mediterranean conditions. *Pedobiologia* **46**, 42–52 (2002).

18. Bonkowski, M., Scheu, S. & Schaefer, M. Interactions of earthworms (*Octolasion lacteum*), millipedes (*Glomeris marginata*) and plants (*Hordelymus europaeus*) in a beechwood on a basalt hill: Implications for litter decomposition and soil formation. *Appl. Soil Ecol.* **9**, 161–166 (1998).

19. Gerlach, A., Russell, D. J., Römbke, J. & Brüggemann, W. Consumption of introduced oak litter by native decomposers (Glomeridae, Diplopoda). *Soil Biol. Biochem.* **44**, 26–30 (2012).

20. Baumeister, N. C. Nutritional ecology of millipedes in pacific northwest conifer forests. (2002).

21. Shaw, G. G. Energy budget of the adult millipede *Narceus annularis*. *Pedobiologia* (1970).

22. Gerlach, A., Russell, D. J., Jaeschke, B. & Römbke, J. Feeding preferences of native terrestrial isopod species (Oniscoidea, Isopoda) for native and introduced leaf litter. *Appl. Soil Ecol.* **83**, 95–100 (2014).

23. HÄttenschwiler, S. & Bretscher, D. Isopod effects on decomposition of litter produced under elevated CO_2_, N deposition and different soil types. *Glob. Change Biol.* **7**, 565–579 (2001).

24. Kheirallah, A. M. Fragmentation of leaf litter by a natural population of the millipede *Julus scandinavius* (Latzel 1884). *Biol.Fert Soils* **10**, 202–206 (2004).

25. Römbke, T., Römbke, J. & Russell, D. Effects of temperature increases on the feeding activity of two species of isopods (*Porcellio scaber, Porcellionides pruinosus)* in laboratory tests. *Soil Org.* **83**, 211–220 (2011).

26. Hashimoto, M., Kaneko, N., Ito, M. T. & Toyota, A. Exploitation of litter and soil by the train millipede Parafontaria laminata (Diplopoda: Xystodesmidae) in larch plantation forests in Japan. *Pedobiologia* **48**, 71–81 (2004).

27. Sousa, J. P., Vingada, J. V, Loureiro, S., da Gama, M. M. & Soares, A. M. V. M. Effects of introduced exotic tree species on growth, consumption and assimilation rates of the soil detritivore *Porcellio dilatatus* (Crustacea: Isopoda). *Appl. Soil Ecol.* **9**, 399–403 (1998).

28. Wood, C. T. & Zimmer, M. Can terrestrial isopods (Isopoda: Oniscidea) make use of biodegradable plastics? *Appl. Soil Ecol.* **77**, 72–79 (2014).

29. Bigler, I., Nentwig, W. & Lindfeld, A. Food preference in the woodlouse Porcellio scaber (Isopoda) in a choice test with fungicidal GM wheat. *J. Appl. Entomol.* **136**, 51–59 (2012).

30. Vilisics, F., Szekeres, S. & Hornung, E. Size dependent differences in litter consumption of isopods: Preliminary results. *ZooKeys* **176**, 247–259 (2012).

31. Dias, N., Hassall, M. & Waite, T. The influence of microclimate on foraging and sheltering behaviours of terrestrial isopods: Implications for soil carbon dynamics under climate change. *Pedobiologia* **55**, 137–144 (2012).

32. Loureiro, S., Sampaio, A., Brandão, A., Nogueira, A. J. A. & Soares, A. M. V. M. Feeding behaviour of the terrestrial isopod *Porcellionides pruinosus* Brandt, 1833 (Crustacea, Isopoda) in response to changes in food quality and contamination. *Sci. Tot. Environ.* **369**, 119–128 (2006).

33. Svyrydchenko, A. O. & Brygadyrenko, V. V. Trophic preferences of *Rossiulus kessleri* (Diplopoda, Julidae) for the litter of various tree species. *Folia Oecologica* **41**, 202–212 (2014).

34. Loginova, N. G. & Busargina, S. A. Feeding activity of the land woodlice, *Trachelipus rathkei* (Oniscoidea), in mountain oak forests of Mordovia. *Zoologicheskiĭ zhurnal* **84**, 1315–1318 (2005).

35. Morón-Ríos, A. Litter consumption by *Xyloryctes lobicollis* (Bates) (Coleoptera: Scarabaeidae: Dynastinae) larvae and its contribution to soil nutrients. *The Coleopterists Bulletin* **62**, 331–332 (2009).

36. Dong, W. & Yin, X. Transformation of carbon and nitrogen by earthworms in the decomposition processes of broad-leaved litters. *Ch. Geogr. Sci.* **17**, 166–172 (2007).

37. Xia, L., Szlavecz, K., Swan, C. M. & Burgess, J. L. Inter- and intra-specific interactions of *Lumbricus rubellus* (Hoffmeister, 1843) and *Octolasion lacteum* (Örley, 1881) (Lumbricidae) and the implication for C cycling. *Soil Biol. Biochem.* **43**, 1584–1590 (2011).

38. Pobozsny, M. Chemische Veränderungen der laubstreu bei der folgezersetzung durch verschiedene bodentiere. (1978).

39. Knollenberg, W. G., Merritt, R. W. & Lawson, D. L. Consumption of Leaf Litter by *Lumbricus terrestris* (Oligochaeta) on a Michigan Woodland Floodplain. *The Amer. Mid. Nat.* **113**, 1–6 (1985).

40. Morales-Ramos, J. & Rojas, M. Growth of young colonies of *Coptotermes formosanus* (Isoptera: Rhinotermitidae) feeding on single versus multiple wood species. *Sociobiology* **46**, 155–173 (2005).

41. Ding, W. & Hu, X. P. Antitermitic effect of the *Lantana camara* plant on subterranean termites (Isoptera: Rhinotermitidae). *Insect Sci.* **17**, 427–433 (2010).

42. Vasconcellos, A. *et al.* Termite assemblages in three habitats under different disturbance regimes in the semi-arid Caatinga of NE Brazil. *J.l of Arid Environ.* **74**, 298–302 (2010).

43. R. B. Barca, R., F. Lucena, E. & Vasconcellos, A. Nest population structure and wood litter consumption by *Microcerotermes indistinctus* (Isoptera) in a Seasonally Dry Tropical Forest, Northeastern Brazil. *Insects* **9**, (2018).

44. Adam, R. A., Mitchell, J. D. & van der Westhuizen, M. C. Aspects of foraging in the harvester termite, *Trinervitermes trinervoides* (Sjöstedt) (Termitidae: Nasutitermitinae). *Afri. Entomol.***16**, 153–161 (2008).
